# NBR1-Mediated Selective Autophagy Targets Insoluble Ubiquitinated Protein Aggregates in Plant Stress Responses

**DOI:** 10.1371/journal.pgen.1003196

**Published:** 2013-01-17

**Authors:** Jie Zhou, Jian Wang, Yuan Cheng, Ying-Jun Chi, Baofang Fan, Jing-Quan Yu, Zhixiang Chen

**Affiliations:** 1 Department of Horticulture, Zhejiang University, Hangzhou, China; 2 Department of Botany and Plant Pathology, Purdue University, West Lafayette, Indiana, United States of America; University of Missouri, United States of America

## Abstract

Plant autophagy plays an important role in delaying senescence, nutrient recycling, and stress responses. Functional analysis of plant autophagy has almost exclusively focused on the proteins required for the core process of autophagosome assembly, but little is known about the proteins involved in other important processes of autophagy, including autophagy cargo recognition and sequestration. In this study, we report functional genetic analysis of Arabidopsis NBR1, a homolog of mammalian autophagy cargo adaptors P62 and NBR1. We isolated two *nbr1* knockout mutants and discovered that they displayed some but not all of the phenotypes of autophagy-deficient *atg5* and *atg7* mutants. Like ATG5 and ATG7, NBR1 is important for plant tolerance to heat, oxidative, salt, and drought stresses. The role of NBR1 in plant tolerance to these abiotic stresses is dependent on its interaction with ATG8. Unlike ATG5 and ATG7, however, NBR1 is dispensable in age- and darkness-induced senescence and in resistance to a necrotrophic pathogen. A selective role of NBR1 in plant responses to specific abiotic stresses suggest that plant autophagy in diverse biological processes operates through multiple cargo recognition and delivery systems. The compromised heat tolerance of *atg5*, *atg7*, and *nbr1* mutants was associated with increased accumulation of insoluble, detergent-resistant proteins that were highly ubiquitinated under heat stress. NBR1, which contains an ubiquitin-binding domain, also accumulated to high levels with an increasing enrichment in the insoluble protein fraction in the autophagy-deficient mutants under heat stress. These results suggest that NBR1-mediated autophagy targets ubiquitinated protein aggregates most likely derived from denatured or otherwise damaged nonnative proteins generated under stress conditions.

## Author Summary

Autophagy is an evolutionarily conserved process that sequestrates and delivers cytoplasmic macromolecules and organelles to the vacuoles or lysosomes for degradation. In plants, autophagy is involved in supplying internal nutrients during starvation and in promoting cell survival during senescence and during biotic and abiotic stresses. Arabidopsis NBR1 is a homolog of mammalian autophagy cargo adaptors P62 and NBR1. Disruption of Arabidopsis *NBR1* caused increased sensitivity to a spectrum of abiotic stresses but had no significant effect on plant senescence, responses to carbon starvation, or resistance to a necrotrophic pathogen. NBR1 contains an ubiquitin-binding domain, and the compromised stress tolerance of autophagy mutants was associated with increased accumulation of NBR1 and ubiquitin-positive cellular protein aggregates in the insoluble protein fraction under stress conditions. Based on these results, we propose that NBR1 targets ubiquitinated protein aggregates most likely derived from denatured and otherwise damaged nonnative proteins for autophagic clearance under stress conditions.

## Introduction

Autophagy is an evolutionary conserved mechanism for degradation of cytoplasmic constituents including proteins and organelle materials [1], [2], [3]. During autophagy, an isolation membrane forms, elongates and sequesters cytoplasmic constituents including organelles. The edges of the membrane then fuse to form a double-membrane vesicle termed autophagosome, which can fuse with the lysosomes or vacuoles to deliver the content for degradation [4]. In the budding yeast, *Saccharomuces cerevisiae*, where autophagy has been well studied, there are more than 30 autophagy-related (ATG) proteins identified [5]. The products of these ATG genes are involved in the induction of autophagy, autophagosome nucleation, elongation, maturation and fusion with vacuoles. Most of the ATG genes initially discovered in yeast have been detected and analyzed in other eukaryotes, suggesting the highly conserved nature of the core autophagy process.

One of the best-characterized and probably most important cellular roles of autophagy is to provide an internal source of nutrients under starvation through nonselective, bulk degradation of cytoplasmic constituents including proteins and organelles [6]. However, autophagy also functions as a quality control mechanism that selectively targets damaged organelles and toxic macromolecules [6]. Selective autophagy is mediated by autophagy adaptors that recognize specific autophagy substrates, on the one hand, and interact with autophagosomal marker protein ATG8, on the other hand, thereby facilitating delivery of captured autophagy cargos to autophagosomes for degradation [6]. In mammalian organisms, for example, autophagic clearance of cytosolic ubiquitinated substrates or aggregate-prone proteins is mediated by autophagy cargo adaptors P62 and NBR1, which bind ubiquitinated proteins via their C-terminal ubiquitin-associated (UBA) domains and the mammalian ATG8 homolog, LC3 (microtubule-associated protein 1 light chain 3) via the LIR (LC3-interacting region) motifs [6]. In addition, Nix acts as an adaptor for autophagy of mitochondria (mitophagy) during erythrocyte differentiation [7]. There are also adaptors for selective autophagy of bacteria and viruses (xenophagy) [8], [9].

Over the past twenty years or so, more than 30 ATG genes have been identified in *Arabidopsis* [10]. Similar ATG genes from other plants including tobacco, rice and maize have also been reported and functionally analyzed [11], [12], [13], [14]. These studies have shown that autophagy plays an important role in nutrient recycling and utilization in plants. Under nitrogen- or carbon-limiting conditions, both the formation of the autophagosome and expression of some of the ATG genes are induced [15], [16]. Furthermore, *Arabidopsis* mutants defective in autophagy are hypersensitive to nitrogen- or carbon-limiting conditions [15], [16], [17], [18], [19]. Apparently, during nutrient deprivation, cells rely on autophagy for degradation of cellular structures or macromolecules for free nutrients and energy in order to survive nutrient starvation. Other studies have revealed that autophagy is also involved in the regulation of plant senescence [19], [20], [21]. Plant senescence can be considered a process of nutrient redistribution. In the parts of plants undergoing senescence such as old leaves, autophagy participates in the degradation of cellular structures and molecules including chloroplasts and chloroplast proteins for efficient nutrient relocalization and utilization by young tissues and developing fruits and seeds.

Autophagy is involved in plant response to biotic stresses. One of the most effective mechanisms in plant immune responses to biotrophic pathogens is immunity-related programmed cell death (PCD) (also known as hypersensitive responses or HR). In *Tobacco mosaic virus* (TMV)-inoculated *Nicotinana benthamiana* expressing the N resistance gene, virus-induced silencing of *ATG6* and *ATG7* genes resulted in expansion of N-mediated HR to uninfected tissue in inoculated leaves and uninfected distant leaves [12]. Likewise, antisence suppression of *Arabidopsis ATG6* limited HR PCD triggered by the *RPM1* R gene in response to the avirulent *Pseudomonas syringae* pv. tomato *DC3000* expressing the avirulent gene *AvrRpm1* [22]. These studies indicate that autophagy negatively regulates HR PCD in plant immune responses to biotrophic pathogens. We recently reported that *Arabidopsis* WRKY33, a transcription factor important for plant resistance to necrotrophic pathogens [23], interacts with an autophagy protein, ATG18a, in the nucleus, suggesting possible involvement of autophagy in plant responses to necrotrophic pathogens [24]. Indeed, autophagy is induced by infection of the necrotrophic fungal pathogen *Botrytis cinerea* and *Arabidopsis* autophagy mutants exhibited enhanced susceptibility to the necrotrophic pathogens *B. cinerea* and *Alternaria brassicicola* [24], [25]. Thus, autophagy plays an important role in plant resistance to necrotrophic fungal pathogens.

Autophagy is also induced in plants during abiotic stresses including oxidative, high salt and osmotic stress conditions [26], [27]. In addition, transgenic RNAi-*AtATG18a* lines defective in autophagy are hypersensitive to ROS, salt and drought conditions [17], [18], [26]. Likewise, rice mutant for *OsATG10b* was hypersensitive to methyl viologen (MV)-induced oxidative stress [13]. Thus, autophagy is involved in in plant responses to a variety of abiotic stresses. Although high temperature is one of the most common abiotic stresses, to our knowledge, there is no reported study that examines the role of autophagy in plant heat tolerance.

Although the roles of autophagy in a wide spectrum of biological processes including stress responses in plants have been well established, our understanding of the mechanistic basis for the important roles of plant autophagy in different biological processes is very limited. Functional analysis of plant autophagy has almost exclusively focused on the genes required for the highly conserved core process of autophagosome formation and it is unclear whether other processes of autophagy such as cargo recognition and delivery operates through the same or different mechanisms in diverse biological processes in plants. To gain knowledge about the mechanistic basis of the roles of autophagy and its regulation in plant defense and stress responses, we were interested in ATG8-interacting autophagy cargo adaptors from plants. Using yeast two-hybrid screens, we isolated several ATG8-interacting proteins from *Arabidopsis* including NBR1, the homolog of mammalian P62 and NBR1 proteins [28]. In the present report, we isolated two independent knockout mutants for *Arabidopsis NBR1* and found that the mutants were compromised in plant tolerance to specific abiotic stresses but were normal in the other biological processes in which autophagy is involved. These results provided genetic evidence that the broad roles of autophagy in diverse biological processes in plants are mediated by multiple, distinct mechanisms. Through a comprehensive molecular, cellular and biochemical analysis of the autophagy mutants, we provided strong evidence that NBR1-mediated autophagy targets ubiquitinated protein aggregates most likely derived from denatured and otherwise damaged nonnative proteins generated under stress conditions.

## Results

### Identification of NBR1 as an ATG8-interacting protein

In Arabidopsis, nine *ATG8* genes (*ATG8a* to *ATG8i*) have been identified [20]. Preliminary qRT-PCR analysis indicated that *ATG8a*, *ATG8e*, *ATG8f* and *ATG8i* were the most abundantly expressed members of the gene family. To identify ATG8-interacting autophagy cargo adaptors using yeast two-hybrid screens, we first fused full-length coding sequences of *Arabidopsis* ATG8a and ATG8f with the DNA-binding domain (BD) of Gal4. Using the fused ATG8 proteins as baits, we screened 4×10^6^ independent transformants of an *Arabidopsis* cDNA prey library and identify more than twenty clones by prototrophy for His and by *Lac*Z reporter gene expression through assays of β-galactosidase activity. The proteins encoded by these positive clones include ATG4 (Ag2g44140), ATI1 (At2g45980) and ATI2 (At4g00355). ATG4, a cysteine protease and known ATG8-interacting protein, cleaves off the C-terminal amino acid of ATG8 to expose a C-terminal glycine residue so it can be conjugated to the lipid phosphatidylethanolamine, a key step in autophagosome formation at the phagophore assembly site [29]. ATI1 and ATI2 are two closed related, plant specific ATG8-interacting proteins that are partially associated with endoplasmic reticulum under normal growth conditions but become mainly associated with newly identified spherical compartments under carbon starvation [30].

Another protein encoded by some of the identified positive clones is NBR1 (At4g24690), the *Arabidopsis* homolog of mammalian P62 and NBR1. In yeast, NBR1 interacted strongly with ATG8f and, to a less extent, with ATG8a, ATG8e and ATG8i. In human, P62, also known as Sequestosome-1 (SQSTM1), acts as an autophagy cargo adaptor by interacting with both ubiquitinated cargo proteins and ATG8, thereby facilitating docking of autophagy substrates to the autophagosomes [6]. There is also growing evidence that human NBR1 cooperates with P62 in the autophagic degradation of ubiquitinated cargo proteins [6]. Mammalian P62 and NBR1 share a number of domains including the N-terminal PB1 domain, a zinc finger domain, a LIR (LC3 or ATG8-interacting) motif and one or two C-terminal UBAs [6]. These conserved domains are also present in *Arabidopsis* NBR1. When our studies on *Arabidopsis* NBR1 were in progress, another group reported the identification and characterization of *Arabidopsis* NBR1 [28]. It was shown that *Arabidopsis* NBR1 homo-polymerized via the PB1 domain and bound ATG8 through its conserved LIR motif [28]. Pull-down assays showed that NBR1 interacted with six of the eight Arabidopsis ATG8 proteins (ATG8e was not tested) [28]. NBR1 did not interact with ATG8h and interacted very weakly with ATG8g [28]. In addition, although *Arabidopsis* NBR1 contains two UBA domains at its C-terminus, only the C-terminal UBA bound ubiquitin [28]. Further analysis using fused fluorescent proteins demonstrated that *Arabidopsis* NBR1 is an autophagy substrate degraded in the vacuole in an autophagy-dependent manner [28]. Identification and characterization of a similar protein from tobacco, Joka2, have also been recently reported [28], [31]. While these results suggest that *Arabidopsis* NBR1 may act as an autophagy cargo adaptor, these authors failed to isolate mutants for *NBR1* and no genetic analysis was performed to determine its biological functions.

**Figure 1:**
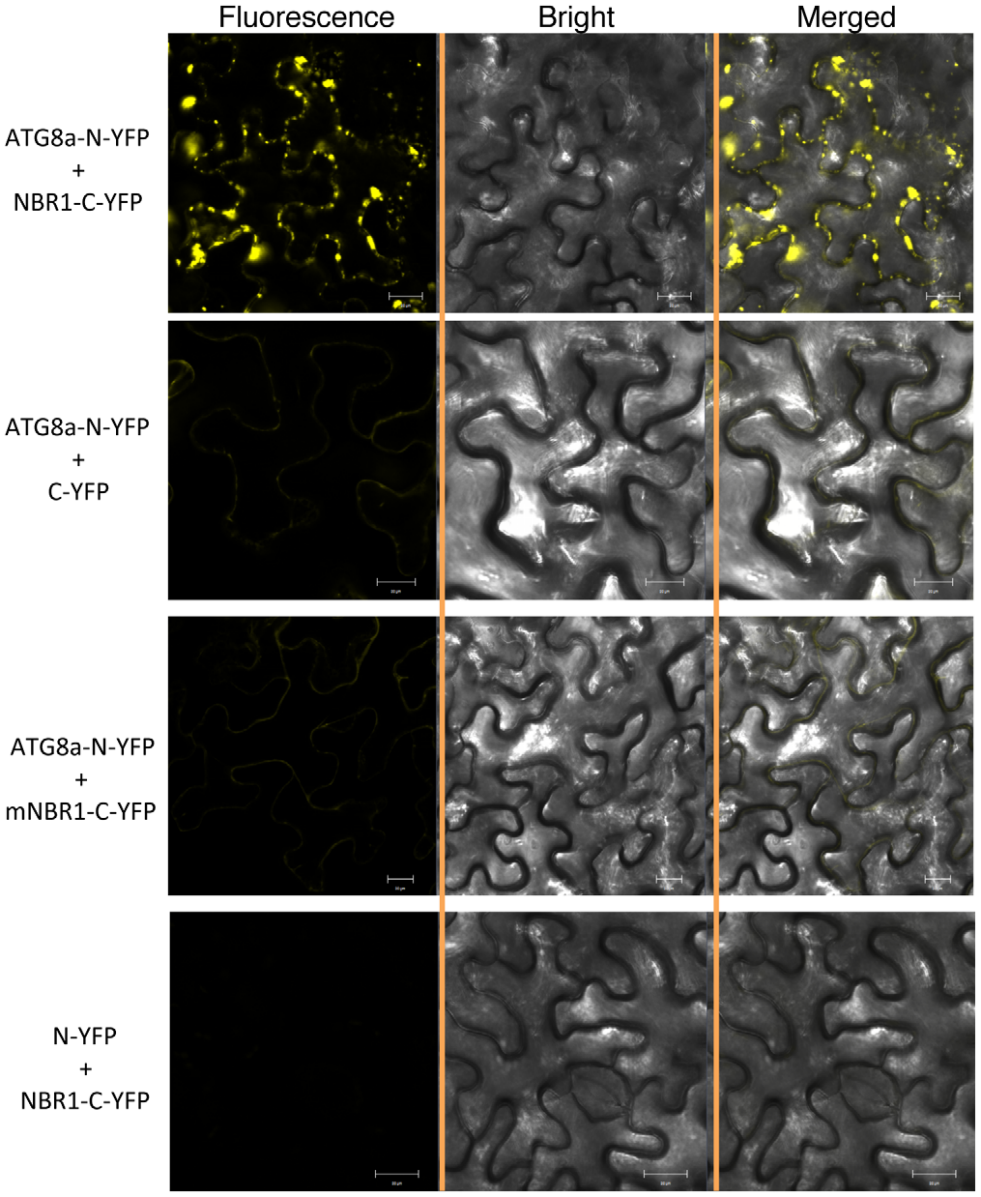
BiFC analysis of NBR1 interaction with ATG8a in planta. Fluorescence was observed in the transformed *N. benthamiana* leaf epidermal cells, which results from complementation of the N-terminal part of the YFP fused with ATG8a (ATG8a-N-YFP) by the C-terminal part of the YFP fused with NBR1 (NBR1-C-YFP). No fluorescence was observed when ATG8a-N-YFP was coexpressed with unfused C-YFP or with mNBR1-C-YFP or when unfused N-YFP was coexpressed with NBR1-C-YFP. YFP epifluorescence images, bright-field images and overlay images of the same cells are shown.

To determine whether ATG8 and NBR1 interact *in vivo*, we performed bimolecular fluorescence complementation (BiFC) in *Agrobacterium tumefaciens*-infiltrated tobacco (*Nicotiana benthamiana*). We fused *Arabidopsis* ATG8a to the N-terminal yellow fluorescent protein (YFP) fragment (ATG8a-N-YFP) and NBR1 to the C-terminal YFP fragment (NBR1-C-YFP). When fused ATG8a-N-YFP was co-expressed with BNR1-C-YFP in tobacco leaves, BiFC signals were detected in transformed cells, including punctate fluorescent structures likely representing pre-autophagosome or autophagosome structures that were induced most likely by bacterial infiltration (Figure 1). Control experiments in which ATG8a-N-YFP was coexpressed with unfused C-YFP or unfused N-YFP was coexpressed with NBR1-C-YFP did not show fluorescence (Figure 1). Furthermore, we generated a mutant NBR1 in which the Trp and Ile residues in the conserved WxxI LIR motif between the two UBA domains were changed to Ala residue (W661A/I664A). Previously it has been shown that the double-point mutant is unable to bind ATG8 *in vitro* [28]. When the mutant NBR1W661A/I664A protein was fused to the C-terminal YFP fragment (mNBR1-C-YFP) and coexpressed with ATG8a-N-YFP, we observed no fluorescence (Figure 1).

### Induction of *ATG* genes and formation of autophagosomes under heat stress

Autophagy is known to play an important role in plant responses to a range of abiotic stresses including salt, drought and oxidative stress. Heat stress due to high temperature is one of the most common abiotic stresses but there is no reported study on the role of autophagy in plant heat tolerance. Heat stress causes accumulation of denatured proteins that are prone to aggregate [32]. In mammalians, P62 and NBR1 are known to target ubiquitinated protein aggregates for selective autophagy [6]. Therefore, we reasoned that NBR1-mediated autophagy could target, among other damaged proteins, heat-denatured proteins and might be necessary for plant heat tolerance.

**Figure 2:**
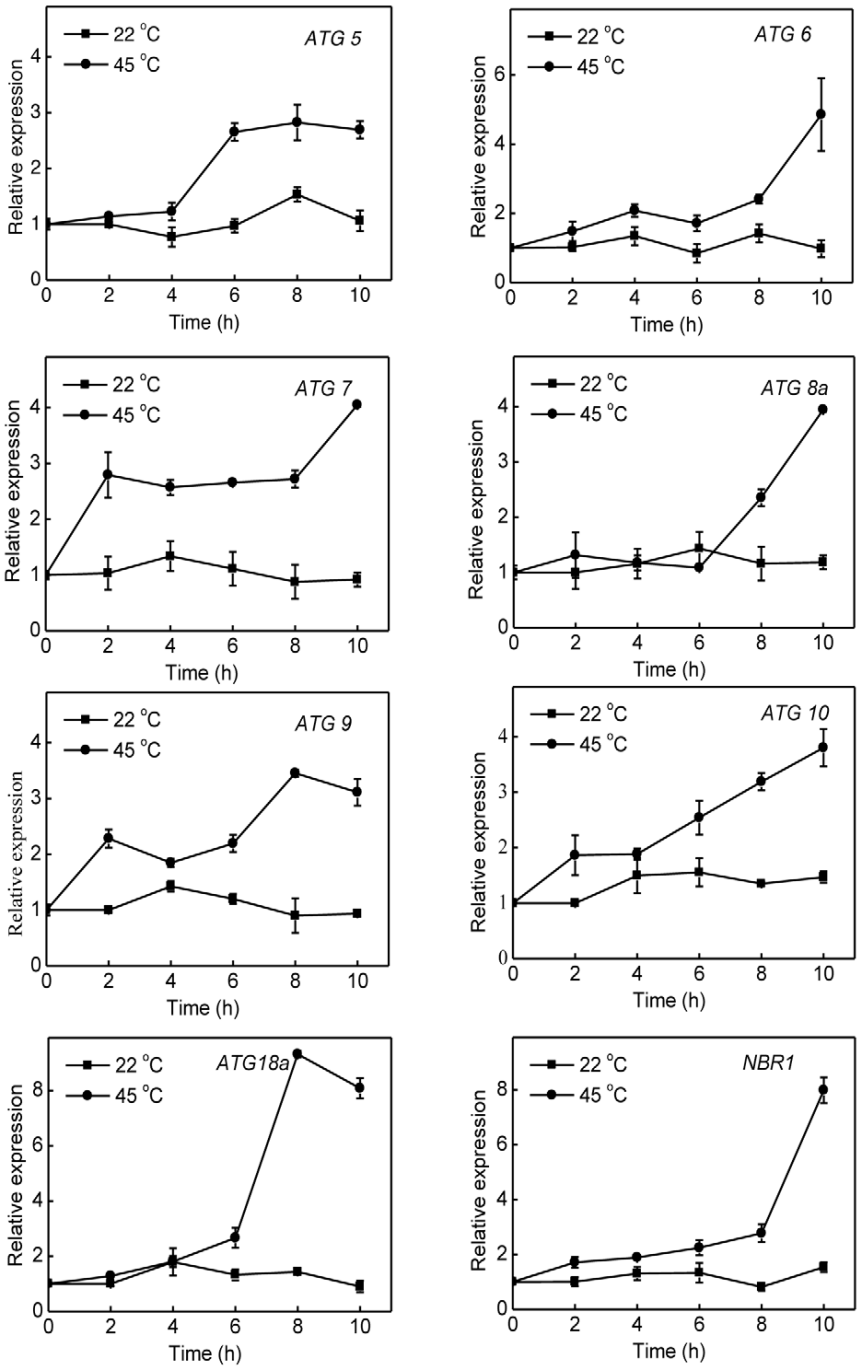
Induction of autophagy genes by heat stress. Five weeks-old *Arabidopsis* wild-type Col-0 plants were placed in the 22°C and 45°C growth chambers and total RNA was isolated from leaf samples collected at indicated times. Transcript levels were determined using real-time qRT-PCR. Error bars indicate SE (n = 3).

To analyze possible involvement of NBR1-mediated autophagy in plant heat tolerance, we first examined the expression patterns of seven *Arabidopsis* autophagy genes (*ATG5*, *ATG6*, *ATG7*, *ATG8a*, *ATG9*, *ATG10*, *ATG18a*) and *NBR1* in response to high temperature. *Arabidopsis* wild-type (Col-0) plants were placed in the 22°C and 45°C chambers and total RNA was isolated from rosette leaves for detection of *ATG* gene transcripts using qRT-PCR. As shown in Figure 2, the transcript levels of the *ATG* and *NBR1* genes remained largely constant throughout the 10-hour period of the experiments at 22°C. At 45°C, however, the transcript levels of the *ATG* genes were elevated with varying kinetics. For some of the *ATG* genes including *ATG7* and *ATG9*, the increased levels of transcripts were detected as early as 2 hours after initiation of the heat stress. Other *ATG* genes including *ATG8a* and *ATG18a* exhibited increased transcript levels after 6-hour exposure to the high temperature (Figure 2). Of note, these *ATG* and *NBR1* genes displayed largest increases in their transcript levels after 8–10 h under heat stress (45°C), when the plants started to show symptoms of dehydration (Figure 2). We also compared induction of the nine members of the *ATG8* gene family and found that there was 4–5 fold induction for *ATG8a*, *ATG8e* and *ATG8h* and 2–3 fold induction for the other members of the gene family following 10-hour heat stress at 45°C (see Figure S1).

**Figure 3:**
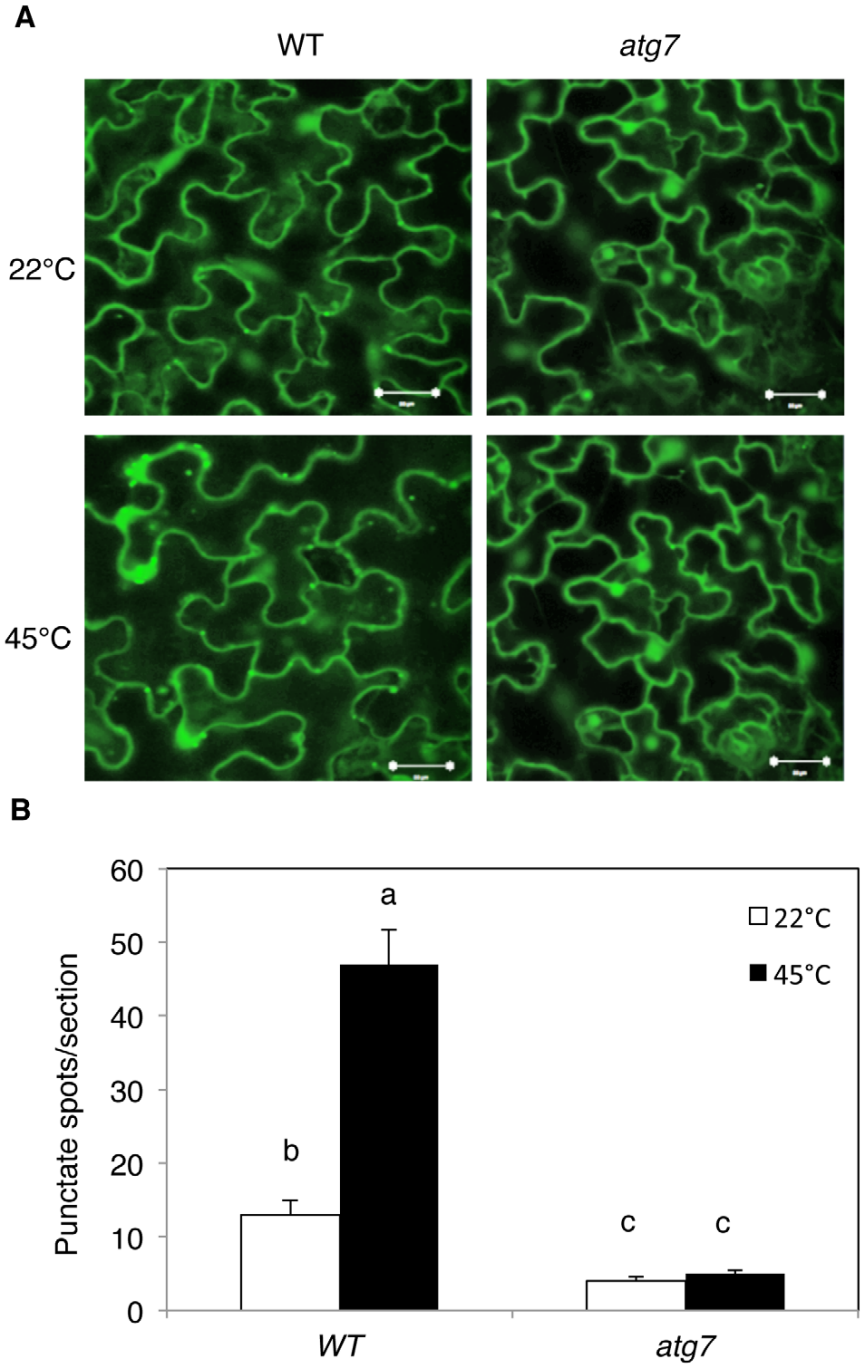
Determination of accumulation of autophagosomes using GFP-ATG8a. (A) Four-weeks old transgenic wild-type Col-0 (WT) and *atg7-2* mutant plants expressing GFP-ATG8a were treated with (45°C) or without (22°C) heat shock for 3 h and then placed at room temperature for 0.5 h. The leaves were visualized by fluorescence confocal microscopy of GFP signal. (B) Numbers of punctate GFP-ATG8a spots representing autophagosomes per 10,000 µm^2^ section. Means and SE were calculated from three experiments. According to Duncan's multiple range test (P = 0.05), means do not differ significantly if they are indicated with the same letter. Bar = 20 µm.

**Figure 4:**
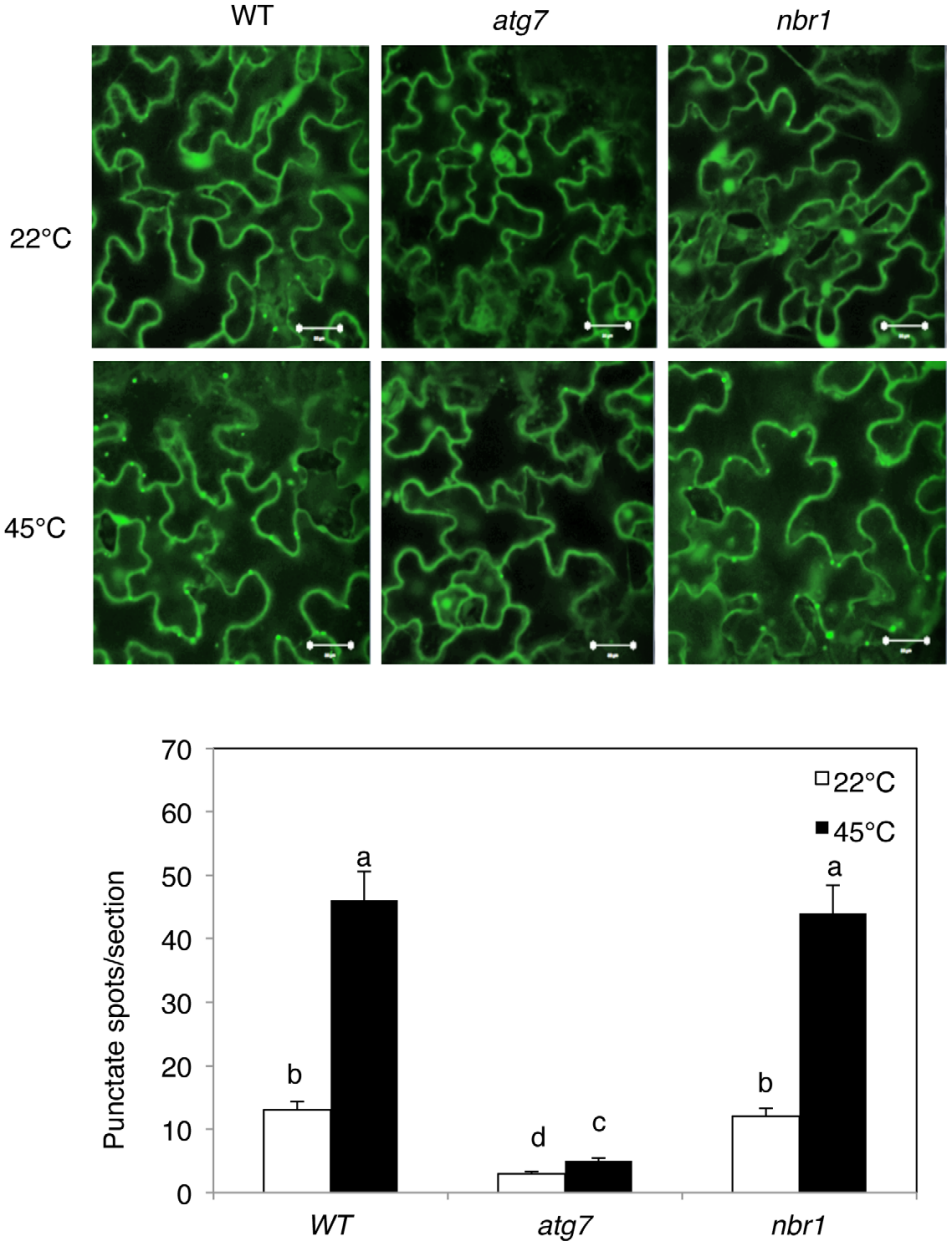
Increased formation of punctate structures containing GFP-NBR1 under heat stress. (Top panel) Four-weeks old transgenic wild-type Col-0 (WT), *atg7-2* and *nbr1-1* mutant plants expressing GFP-NBR1 were treated with (45°C) or without (22°C) heat shock for 3 h and then placed at room temperature for 0.5 h. The leaves were visualized by fluorescence confocal microscopy of GFP signal. (Bottom panel) Numbers of punctate GFP-NBR1 spots per 10,000 µm^2^ section. Means and SE were calculated from three experiments. According to Duncan's multiple range test (P = 0.05), means do not differ significantly if they are indicated with the same letter. Bar = 20 µm.

To further assess induction of autophagy by heat stress, we examined the effect of heat stress on induction of autophagosome formation using green fluorescent protein (GFP)-tagged ATG8a, which is associated with autophagosomes and therefore can be used as a marker of autophagosomes in *Arabidopsis* [15], [33], [34]. Transgenic plants expressing GFP-ATG8a were exposed to 45°C for 3 h, recovered for 0.5 h at room temperature and then observed by confocal fluorescence microscopy. In the wild-type Col-0 background, we observed a low number of punctate GFP signals in the plants grown at 22°C (Figure 3). In heat-treated Col-0 plants, there was a 3-fold increase in punctate GFP-ATG8a fluorescent structures likely representing pre-autophagosome or autophagosome structures (Figure 3). In the *atg7* mutant background, GFP-ATG8a signal was observed but there were few punctate structures (Figure 3). We also generated transgenic *NBR1-GFP* plants and observed an increase in punctate NBR1-GFP signals in wild-type and *nbr1* mutant plants but not in the *atg7* mutant background under heat stress (Figure 4). Expression of *NBR1-GFP* complemented the *nbr1* heat sensitive mutant phenotypes (data not shown). Thus, both expression of *ATG* genes and formation of autophagosomes were induced under heat stress.

### Compromised phenotypes of *atg* and *nbr1* mutants in heat tolerance

To provide genetic analysis of the role of *Arabidopsis* NBR1, we screened five independent T-DNA insertion stocks and isolated a T-DNA insertion mutant for *Arabidopsis NBR1* (*nbr1-1*) from one of them. The *nbr1-1* mutant contains a T-DNA insertion in the fourth exon and had only about 3% of the wild-type level of *NBR1* transcript, indicating that it is likely to be a knockout mutant (see Figure S2). Like other autophagy mutants, *nbr1-1* mutant plants were normal in growth and development and displayed no detectable morphological phenotypes. We, therefore, used the mutant to analyze the role of *NBR1* in the biological processes in which autophagy is involved.

**Figure 5:**
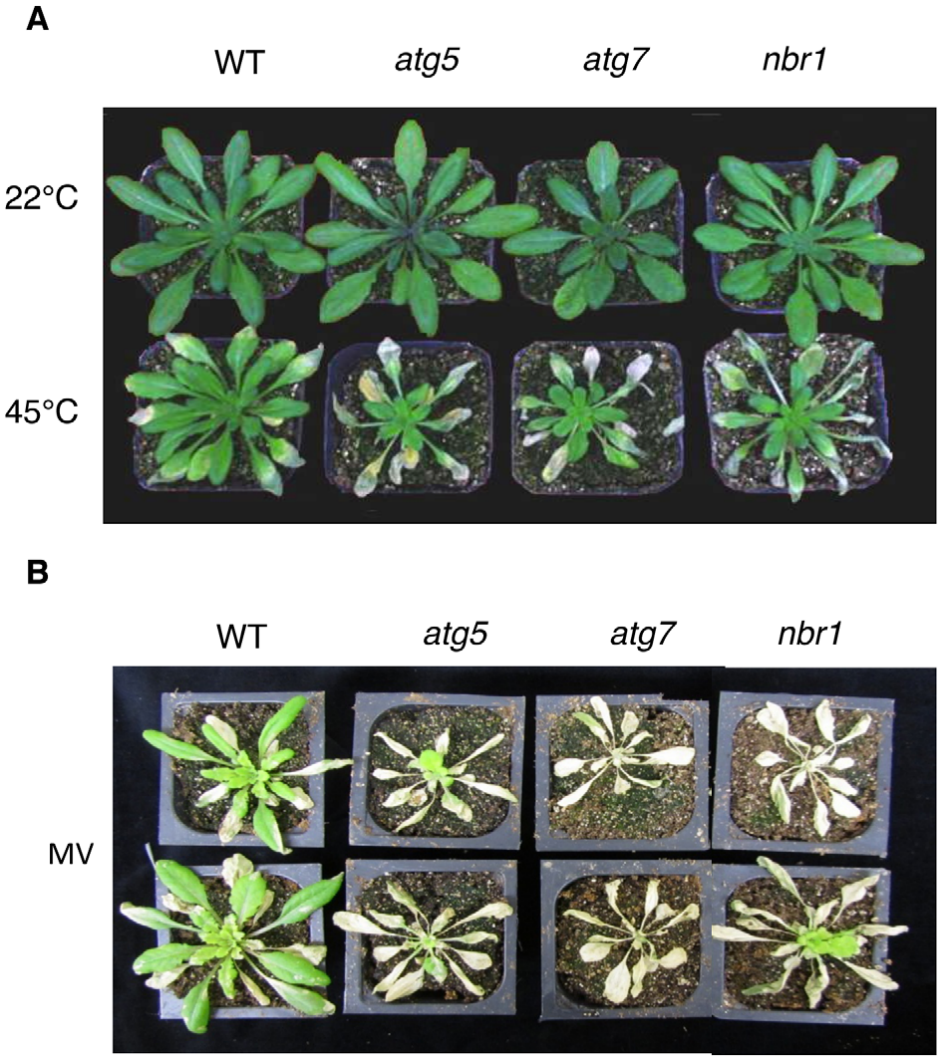
Enhanced sensitivity of *atg5*, *atg7* and *nbr1* mutants to heat and oxidative stresses. (A) Five weeks-old *Arabidopsis* Col-0 wild type (WT), *atg5*, *atg7* and *nbr1* mutant plants were placed in 22°C and 45°C growth for 10 hours and then moved to room temperature for 3-day recovery or (B) sprayed with 20 µM methyl viologen (MV) and kept under light for two days before the picture of representative plants was taken. The experiments were repeated three times with similar results.

First, to analyze directly the role of NBR1-mediated autophagy in heat tolerance, we compared Col-0 wild type, *atg5-1*, *atg7-2* and *nbr1-1* to heat stress. The wild type and mutants were placed in a 45°C growth chamber for 10 hours followed by 3–5 days of recovery at the room temperature. For heat-treated wild-type plants, only some patches of old leaves displayed symptoms of dehydration while a majority of the leaves remained green and viable after the recovery (Figure 5A). On the other hand, a majority of leaves from the *atg* and *nbr1* mutant plants exhibited extensive wilting and bleaching after the recovery (Figure 5A). Thus, disruption of the *ATG* or *NBR1* gene caused increased sensitivity to heat stress.

**Figure 6:**
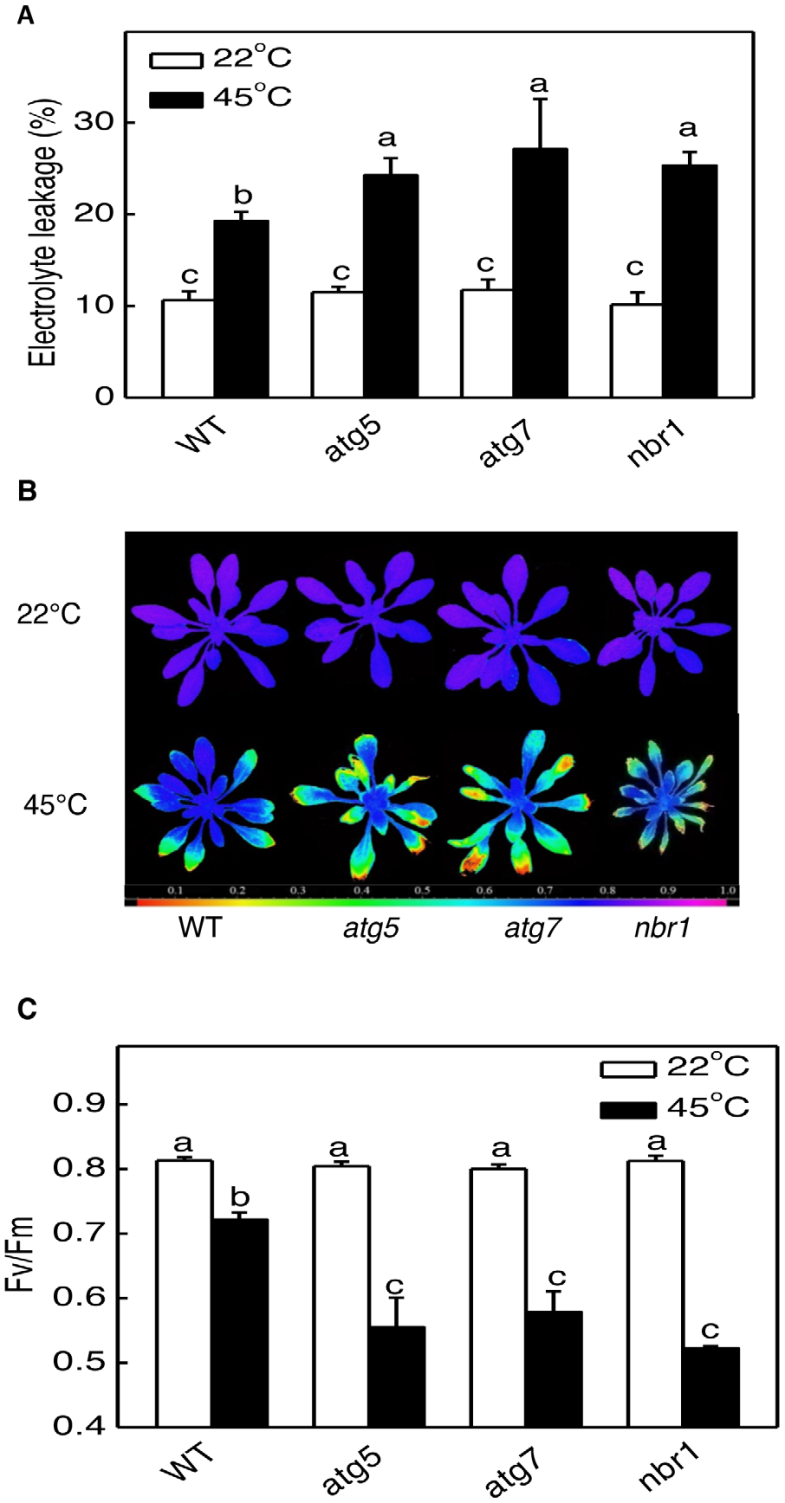
Enhanced sensitivity of *atg5*, *atg7*, and *nbr1* mutants to heat stress. (A) Electrolyte leakage. (B) *Fv/Fm* images. (C) Average values for the *Fv/Fm* images. Five weeks-old *Arabidopsis* Col-0 wild type (WT), *atg5*, *atg7* and *nbr1* mutant plants were placed in 22°C and 45°C growth chambers. Fully expanded leaves were sampled after 10 hours in the indicated temperatures and immediately measured for electrolyte leakage and *Fv/Fm*. Error bars in (A) and (C) indicate SE (n = 5). According to Duncan's multiple range test (P = 0.05), means of EL or *Fv/Fm* do not differ significantly if they are indicated with the same letter. The experiments were repeated twice with similar results.

At biochemical levels, heat stress causes increased membrane permeability, aggregation of the light-harvesting complex of photosystem II (PSII) and inhibition of PSII [35], [36], [37]. Therefore, we also compared the mutants with the wild type for difference in the electrolyte leakage (EL) and maximum quantum yield of PSII (*F*v/*F*m) of fully expanded leaves immediately after heat treatment. As shown in Figure 6, both the EL and *F*v/*F*m values of these mutants were similar to those of wild-type plants when they were grown at 22°C. After 10-h heat stress at 45°C, the EL values for *atg5-1, atg7-2 and nbr1* mutants were 26, 41 and 40% higher than that of wild type, respectively (Figure 6A). Likewise, heat stress caused ∼30% more reduction in *F*v/*F*m in the *atg5, atg7* and *nbr1* mutants than in the wild-type plants (Figure 6B and 6C). Thus, membrane integrity and the capacity of PSII photochemistry were more compromised in the *atg* and *nbr1* mutants than in wild type. We subsequently isolated a second mutant for NBR1 (*nbr1-2*) that contains a T-DNA insertion in the third exon and had only about 5% of the wild-type level of *NBR1* transcript (Figure S2). The *nbr1-2* mutant was equally sensitive to heat stress as *nbr1-1* mutant (data not shown). In addition, as described below, transformation of the wild-type *NBR1* gene into the *nbr1-1* mutant restored the heat tolerance in the mutant. These results indicated that the mutant phenotype of compromised heat tolerance of *nbr1-1* is due to disruption of *NBR1*.

### Compromised phenotypes of *nbr1* mutants in tolerance to oxidative, drought, and salt stresses

**Figure 7:**
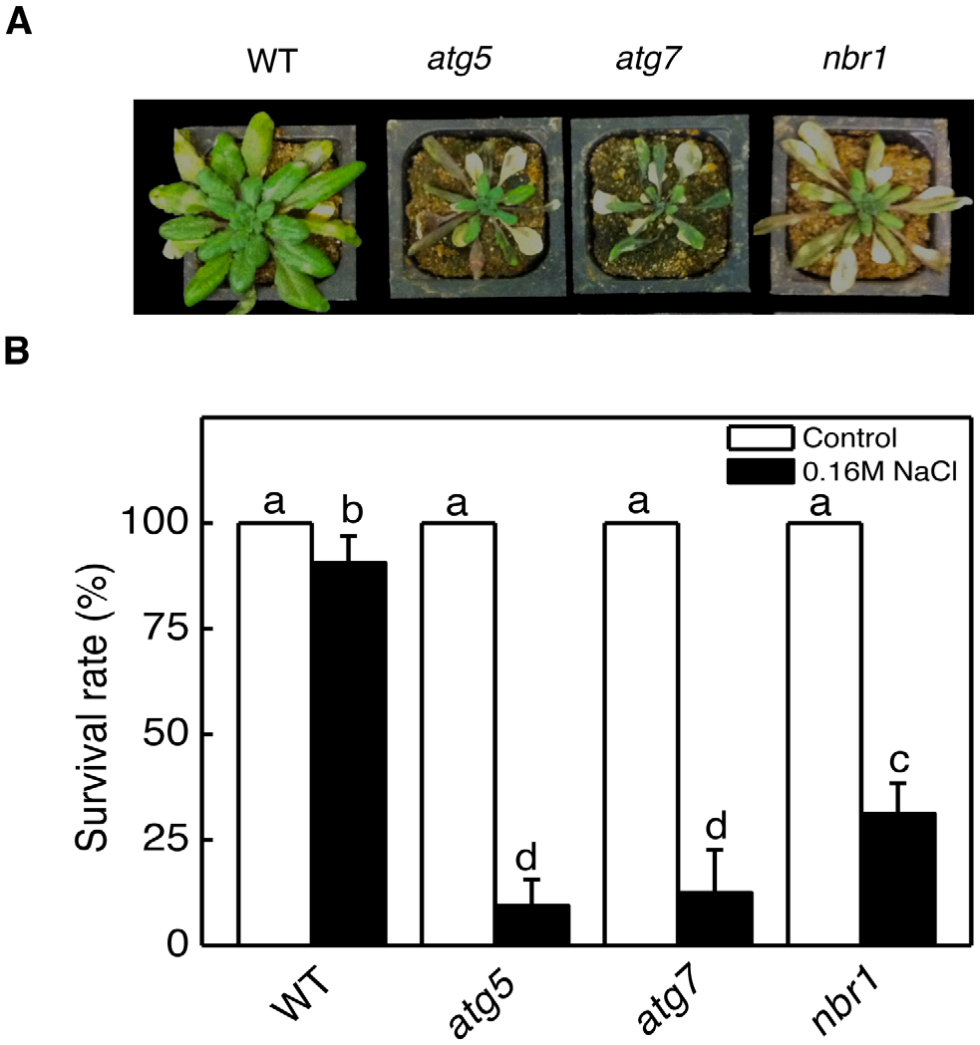
Enhanced sensitivity of *atg5*, *atg7*, and *nbr1* mutants to drought and salt stresses. (A) Five weeks-old *Arabidopsis* Col-0 wild type (WT), *atg5*, *atg7* and *nbr1* mutant plants were placed into a growth chamber with approximately 50% humidity. The photograph of representative plants was taken 10 days after withholding watering. The experiment was repeated twice with similar results. (B) Seven days-old seedlings Col-0 WT, *atg5*, *atg7* and *nbr1* grown on solid MS medium were transferred to the same medium (control) or the same medium containing 0.16 M NaCl and photographed 5 days later. The survived seedlings were scored 5 days after the transfer and the average values and SE were calculated from three experiments. According to Duncan's multiple range test (P = 0.05), means do not differ significantly if they are indicated with the same letter.

Autophagy is known to play an important role in plant responses to oxidative, salt and osmotic stress. When five-weeks old wild type, *atg5*, *atg7* and *nbr1* mutants were sprayed with 20 µM MV, a ROS-generating herbicide, and kept under light for two days, old leaves were bleached but more than 80% of leaf areas remained green in wild-type plants (Figure 5B). By contrast, 80–100% of leaf areas of the *atg5*, *atg7* and *nbr1* mutant plants were bleached after MV treatment (Figure 5B). Thus, like autophagy mutants, *nbr1* mutant plants were hypersensitive to oxidative stress. For testing drought tolerance, we transferred five-weeks old *Arabidopsis* plants into a growth chamber with ∼50% humidity. The plants were unwatered and observed for drought stress symptoms. As shown in Figure 7A, wild-type plants were still largely green and exhibited relatively minor wilting 10 days after watering was stopped. The *atg* and *nbr1* mutants, on the other hand, showed extensive wilting and drought stress symptoms (Figure 7A). For testing salt tolerance, seven days-old seedlings grown on solid MS medium were transferred to the same medium with or without addition of 0.16 M NaCl and the survived seedlings were scored 5 days after the transfer. As shown in Figure 7B, ∼90% of wild-type seedling survived in the medium containing 0.16 M NaCl. On the other hand, only about 10–15% of *atg5*, *atg7* and 35% of *nbr1* mutant seedlings survived in the salt medium (Figure 7B). Thus, like autophagy-deficient *atg* and *atg7*, *nbr1* was sensitive to oxidative, drought and salt stresses.

### Normal phenotypes of *nbr1* in age- and darkness-induced senescence and disease resistance

**Figure 8:**
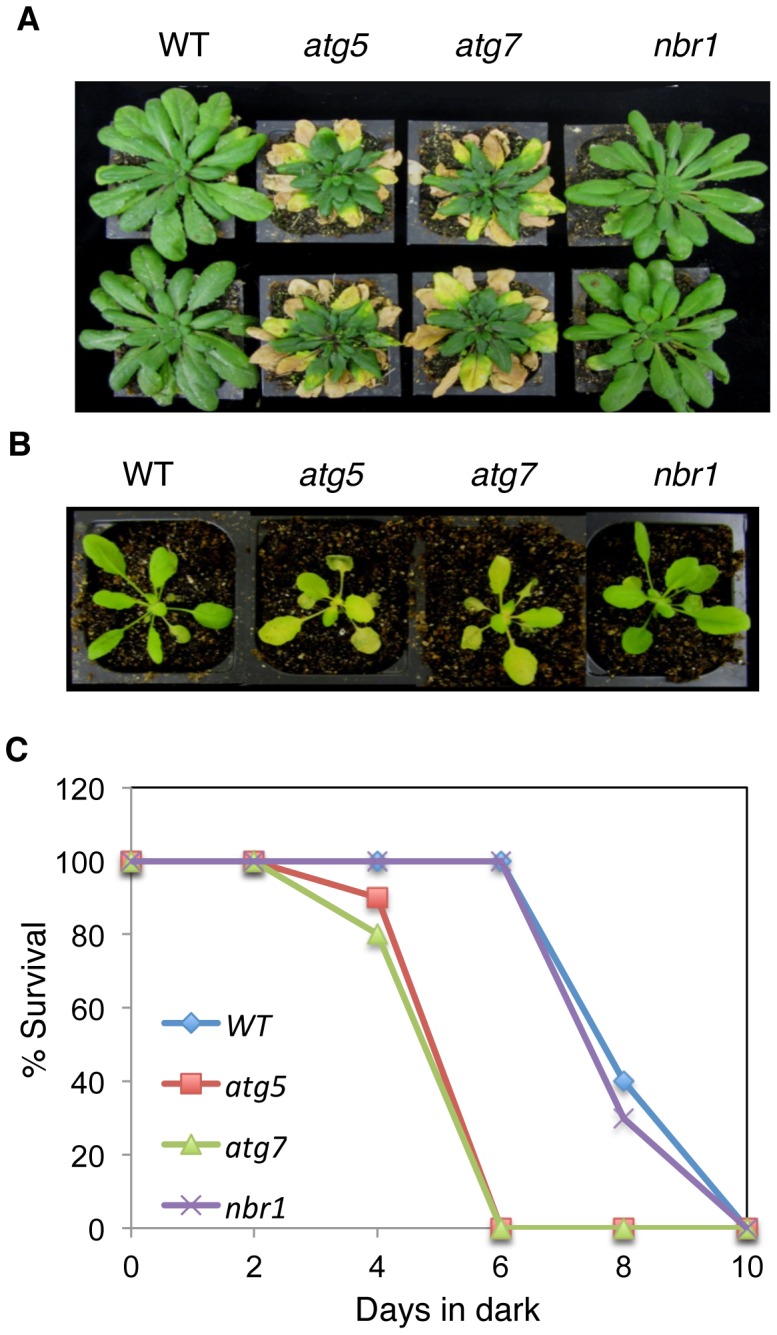
Normal phenotypes of the *nbr1* mutant plants in age- and darkness-induced senescence. (A) Eight weeks old wild type (WT), *atg5*, *atg7* and *nbr1* plants grown under normal growth conditions in a growth chamber. (B) Four weeks old WT, *atg5*, *atg7* and *nbr1* plants after being kept in the dark for 6 days. (C) Percentage of plants that survived the various lengths in dark as determined by resumption of growth. Each point represents the average of 10 plants. The experiments were repeated twice with similar results.

Autophagy is also involved in the regulation of plant senescence, response to nutrient deprivation and resistance to necrotrophic pathogens. As an autophagy receptor, NBR1 might play a broad role in autophagy that recognizes and facilitates docking of a wide range of cellular structures and proteins or target damaged proteins generated by specific stress conditions to the autophagosomes for autophagic clearance. To distinguish between these two possibilities, we also compared Col-0 wild type, *nbr1* and autophagy *atg5* and *atg7* mutants for phenotypes in senescence, response to nutrient deprivation and disease resistance. As reported previously [38], *atg5* and *atg7* mutants were indistinguishable from wild type during the first 4–5 weeks after germination when grown under normal conditions but displayed enhanced senescence afterwards as indicated from increased chlorosis of old, fully-expanded leaves (Figure 8A). The *nbr1* mutant plants, on the other hand, exhibited no significant difference from wild-type plants in the timing or extent of age-related senescence (Figure 8A). Thus, unlike *atg5* and *atg7*, *nbr1* is normal in age-induced senescence. To determine whether the *nbr1* is compromised in response to carbon-deprivation, we placed the *nbr1* mutant plants in the dark along with wild type and *atg5* and *atg7* mutants. As shown in Figure 8B, after 5 days in dark, the *atg5* and *atg7* mutants started to display severe chlorosis but the *nbr1* mutant plants were as green as wild-type plants (Figure 8B). When plants were kept in dark for 6 days and then returned to light, both the *atg5* and *atg7* mutants had a drastic reduction in survival rates while all of the wild type and *nbr1* mutant plants recovered (Figure 8C). These results indicated that the *nbr1* mutant was not compromised in response to carbon-starvation.

**Figure 9:**
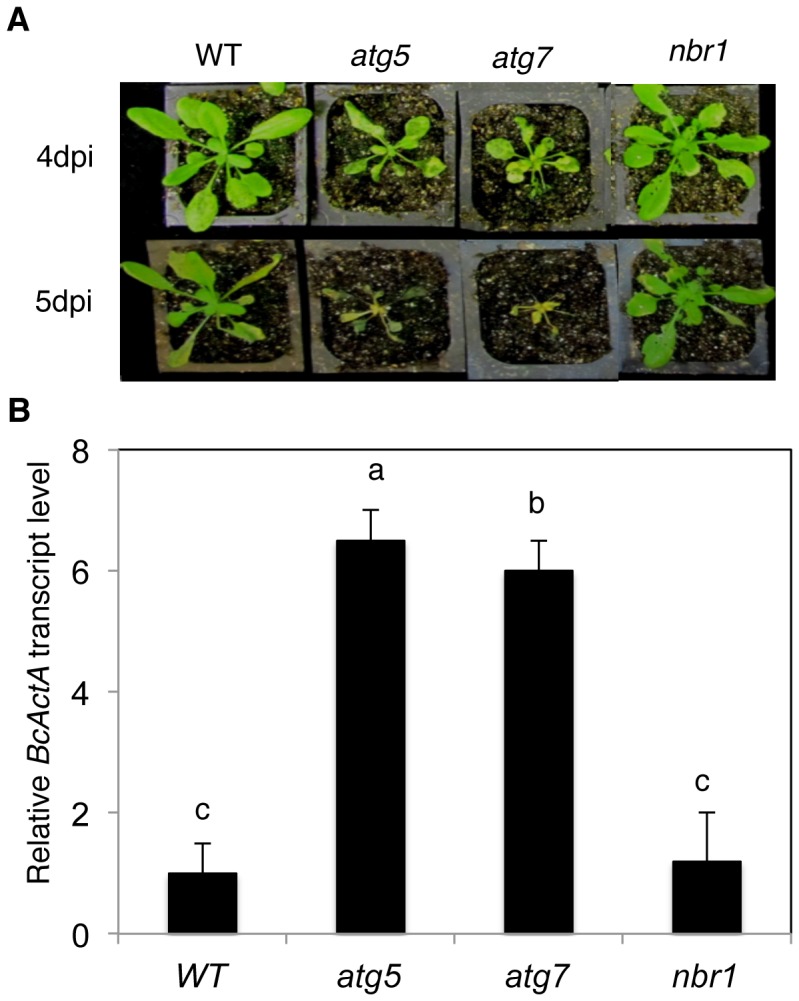
Normal phenotypes of the *nbr1* mutant plants in resistance to *Botrytis*. (A) Responses of Col-0 wild type (WT), *atg5*, *atg7* and *nbr1* plants to *Botrytis*. Wild-type and mutant plants were inoculated by spraying with spore suspension at a density of 2.5×10^5^ spores ml^−1^, and kept at high humidity. Pictures of representative plants were taken at 4 and 5 days post inoculation (dpi). (B) Quantitative real-time PCR analysis of the *B. cinerea ActA* (*BcActA*) transcript levels in infected Arabidopsis plants at 5 dpi. The experiments were repeated twice with similar results.

We have recently reported that *atg* mutants were susceptible to necrotrophic fungal pathogens [24]. To assess the involvement of NBR1 in plant responses to necrotrophic fungal pathogens, we compared the *nbr1* mutant with Col-0 wild type, *atg5* and *atg7* mutants for resistance to the necrotrophic fungal pathogen *B. cinerea*. In the *atg5* and *atg7* mutants, the necrotic spots and chlorosis spread rapidly, and the majority of leaves displayed chlorosis by 4 dpi, and macerated by 5 dpi (Figure 9A). By contrast, the majority of leaves from wild type and *nbr1* remained green at 4 dpi and 5 dpi (Figure 9A). Quantitative real-time PCR (qRT-PCR) indicated that *B*. *cinerea ActA* gene transcript levels in the *atg5* and *atg7* mutants were 6–8 times higher than those in the wild type and *nbr1* mutant plants (Figure 9B). Thus, both disease symptoms and fungal growth indicated that the *nbr1* mutant was not compromised in resistance to the necrotrophic pathogen.

### The role of NBR1 in stress tolerance is dependent on interaction with ATG8

**Figure 10:**
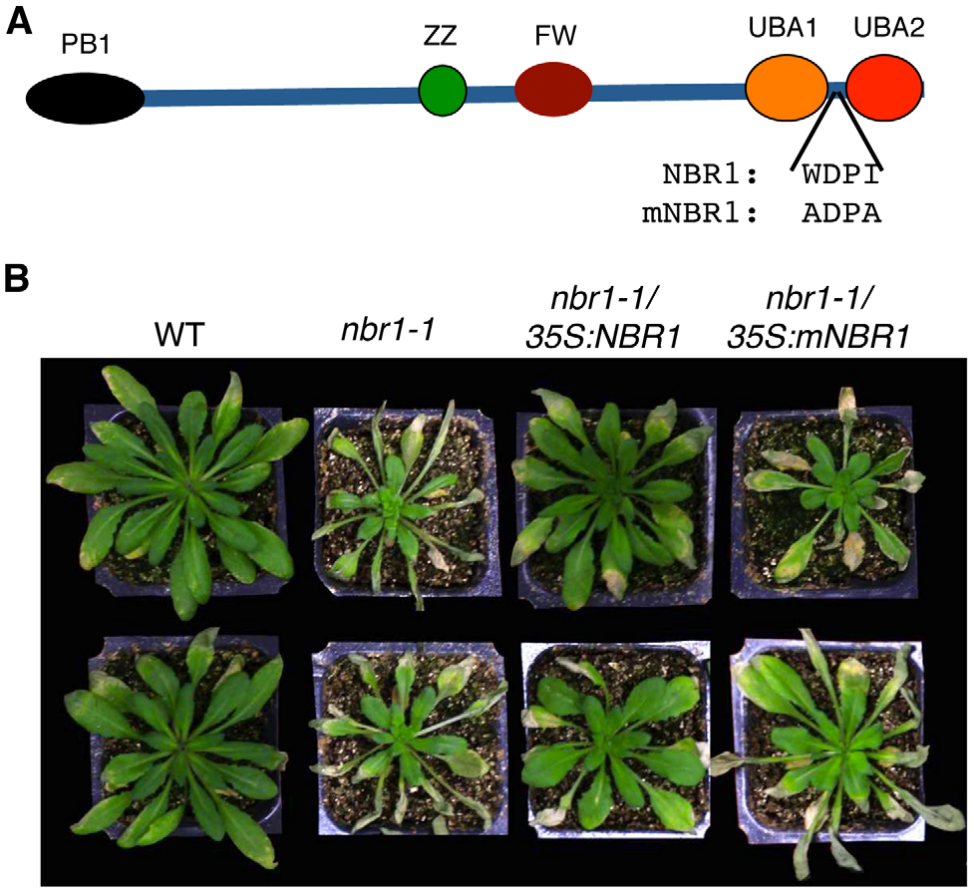
Requirement of the LIR motif for the critical role of NBR1 in heat tolerance. (A) Diagrams of wild-type and mutant NBR1 proteins. The conserved W and I residues in the LIR motif between the two UBA domains were changed to A residues in the NBR1W661A/I664A (mNBR1) mutant protein. (B) Five weeks-old *Arabidopsis* Col-0 wild type (WT), *nbr1* and transgenic *nbr1* plants expressing the wild-type *NBR1* (lines 3 and 6; see Figure S2) or the *NBR1W661A/I664A* (*mNBR1*) (lines 2 and 6; see Figure S2) mutant gene were placed in a 45°C growth chambers for 10 hours and then moved to room temperature for 3-day recovery before the picture as taken. The experiment was repeated three times with similar results.

To determine whether the role of NBR1 in plant stress tolerance is due to its action as an autophagy cargo adaptor that interacts with ATG8, we performed genetic complementation of *nbr1-1* with *NBR1* genes that differ in the LIR motif for interaction with ATG8. We generated a mutant NBR1 in which the Trp and Ile residues in the conserved WxxI LIR motif between the two UBA domains were changed to Ala residue (W661A/I664A) (Figure 10A). The double-point mutant is unable to bind ATG8 *in vitro* [28] or *in vivo* (Figure 1). Both the wild-type and W661A/I664A mutant *NBR1* genes, tagged with a myc epitope, were placed into a plant transformation vector under the control of the *CaMV 35S* promoter and transformed into *nbr1-1*. Transgenic plants were first analyzed by western blotting using anti-myc antibody (see Figure S3) and those plants with similar levels of tagged NBR1 proteins were identified and tested for heat stress tolerance (Figure S3). As expected, the *nbr1-1* mutant was compromised in heat tolerance as indicated from enhanced symptoms developed after heat stress (Figure 10B). Transformation of *nbr1-1* with the wild-type *NBR1* gene completely restored the heat tolerance of the mutant (Figure 10B). In contrast, in the transgenic *nbr1* mutant plants expressing the gene for mutant NBR1 W661A/I664A, there was no restoration of heat tolerance as indicated from the severe symptoms developed after heat treatment (Figure 10B). Likewise, transformation of the *nbr1* mutant with the wild-type *NBR1* gene but not the mutant *NBR1 W661A/I664A* gene restored the tolerance of the mutant to PQ-induced oxidative stress (data not shown). These results indicated that interaction with ATG8 is necessary for the important role of NBR1 in plant stress tolerance.

### Association of heat sensitivity and accumulation of ubiquitin-positive, detergent-resistant protein aggregates

**Figure 11:**
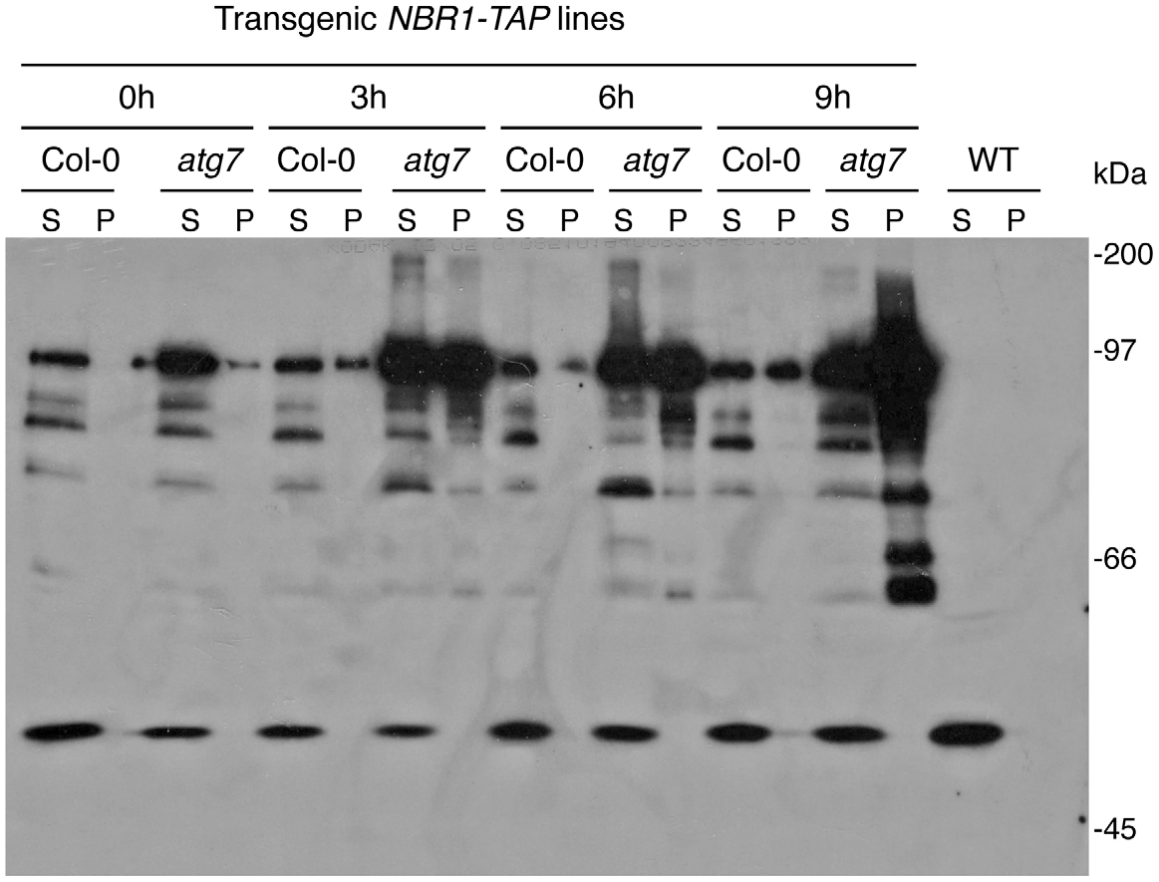
Accumulation of NBR1 and its increased enrichment in the insoluble protein fraction in the *atg7* mutant under heat stress. Leaf tissues from wild type (WT) and *atg7* expressing NBR1-TAP were collected at indicated hours (h) under 45°C and prepared for soluble and insoluble proteins as described in Materials and Methods. Proteins from the first supernatants (S) and last pellets (P) were subjected to SDS-PAGES and probed with a peroxidase-conjugated anti-peroxidase antibody for detection of NBR1-TAP. Protein samples prepared from non-transgenic Col-0 wild-type plants were included as controls.

As an autophagy adaptor, NBR1's role in plant tolerance to specific abiotic stresses is likely to be mediated by its ability in capturing and delivering specific autophagy cargos to autophagosomes for degradation. To identify NBR1-recognized autophagy cargos, we constructed transgenic lines in both Col-0 and *atg7* mutant backgrounds that harbor tandem-affinity purification (TAP)-tagged NBR1 under control of the constitutive *CaMV 35S* promoter. Transgenic lines containing similar levels of *NBR1-TAP* transgene transcripts were identified by RNA blotting (Figure S4) and used for purification of NBR1-interacting proteins. After heat stress, total soluble proteins were isolated from the transgenic plants and subjected to the TAP procedure [39]. However, even in the heat-stressed *atg7* mutant background, in which the autophagy cargo proteins are expected to accumulate, several attempts of affinity purification of NBR1-containing protein complexes from the soluble protein fraction failed to isolate NBR1-interacting proteins that could be readily and reproducibly detected by Coomassie blue staining following sodium dodecyl sulfate polyacrylamide gel electrophoresis (SDS/PAGE). The failure to isolate cargo proteins of NBR1 from the soluble protein fraction prompted us to examine NBR1-TAP and associated proteins in the insoluble protein faction. After homogenization in a detergent-containing buffer and filtering through nylon membranes, insoluble detergent-resistant protein aggregates were separated from soluble proteins by low-speed centrifugation [40]. Both soluble proteins in the supernatant and insoluble proteins in pellets were separated by SDS/PAGE and NBR1-TAP was detected by western blotting. As shown in Figure 11, in transgenic wild-type plants grown at 22°C, NBR1-TAP was detected almost exclusively in the supernatant. Under heat stress at 45°C, some NBR1-TAP was also detected in the insoluble pellet but there was only marginal change in the total levels of NBR1-TAP in the transgenic wild-type plants (Figure 11). In the transgenic *atg7* mutant background, a majority of NBR1-TAP was also present in the supernatant when grown under 22°C (Figure 11). Interestingly, the levels of NBR1-TAP in the transgenic *atg7* mutant plants increased substantially after 3–6 hours at 45°C and there was an increased enrichment of NBR1-TAP in the insoluble detergent-resistant pellets in heat-stressed *atg7* mutant (Figure 11). After 9 hours under 45°C, a majority of NBR1-TAP in the transgenic *atg7* plants was detected in the pellets (Figure 11). Thus, in the autophagy-deficient mutant, heat stress resulted in increased accumulation of NBR1 as insoluble protein aggregates.

**Figure 12:**
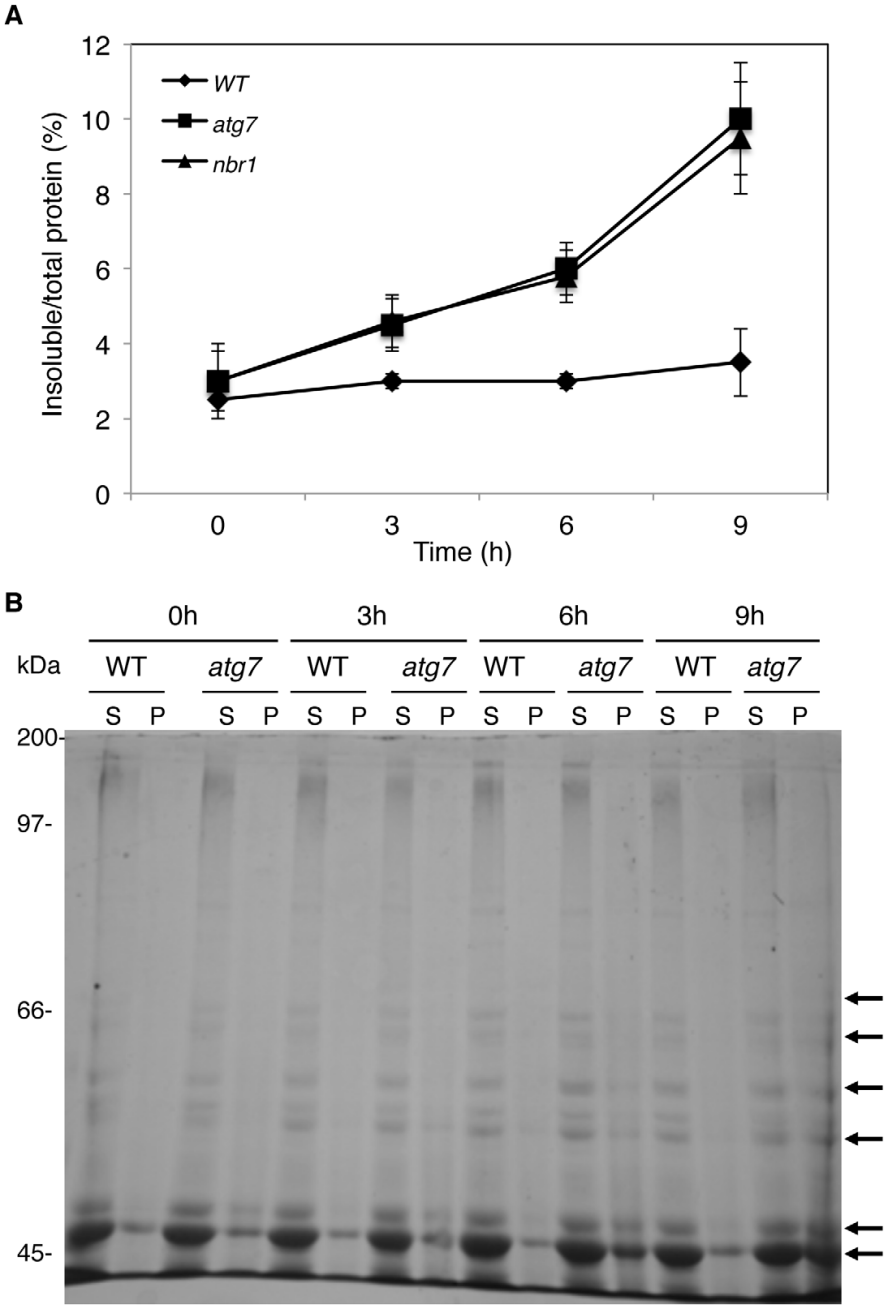
Increased accumulation of insoluble protein aggregates in the *atg7* and *nbr1* mutants under heat stress. (A) Accumulation of insoluble proteins. Leaf tissues from wild-type (WT), *atg7* and nbr1 mutants collected at indicated hours (h) under 45°C for preparation of total, soluble and insoluble proteins as described in Materials and Methods. Total proteins in the starting homogenates and insoluble proteins in the last pellets were determined the percentages of insoluble proteins to total proteins were calculated. (B) Profiles of soluble and insoluble proteins. Proteins from the first supernatants (S) and last pellets (P) were subjected to SDS-PAGES and stained with Coomassie brilliant blue. Major proteins accumulated in the pellets from heat-stressed *atg7* mutant plants were indicated by arrows.

The increased accumulation of NBR1-TAP in the insoluble fraction and the failure to isolate NBR1-interacting proteins from the soluble fraction in heat-stress *atg7* mutant plants raised the possibility that the cargo proteins of NBR1 might be predominantly present in the pellets as insoluble protein aggregates. To test this possibility, we directly compared wild type, *atg7* and *nbr1* mutants for heat-induced accumulation of insoluble detergent-resistant protein aggregates. The plants were first subjected to various periods of 45°C heat stress and soluble and insoluble proteins were quantitated after separation by low-speed centrifugation. As shown in Figure 12A, the percentages of insoluble to total proteins in the wild type did not display significant change over the period of 9 hours under heat stress (Figure 12A). By contrast, in both the *atg7* and *nbr1* mutants, insoluble proteins increased significantly after 3-hour exposure to 45°C (Figure 12A). After 9 hours at 45°C, the levels of insoluble protein aggregates in the *atg7* and *nbr1* mutants were about three times of those in the wild type (Figure 12A). To compare the profiles of the insoluble proteins with those of soluble ones, we separated the proteins on SDS/PAGE. As shown in Figure 12B, increased levels of insoluble proteins in heat-stressed *atg7* and *nbr1* mutants were associated with the detection of major protein bands on SDS/PAGE gel that appeared to be the same proteins as the the most abundant proteins in the soluble fraction based on the migration patterns (indicated by arrows in Figure 12B). Thus, the most abundant insoluble proteins accumulated in the heat-stressed autophagy mutants were likely to be derived from the most abundant soluble proteins.

**Figure 13:**
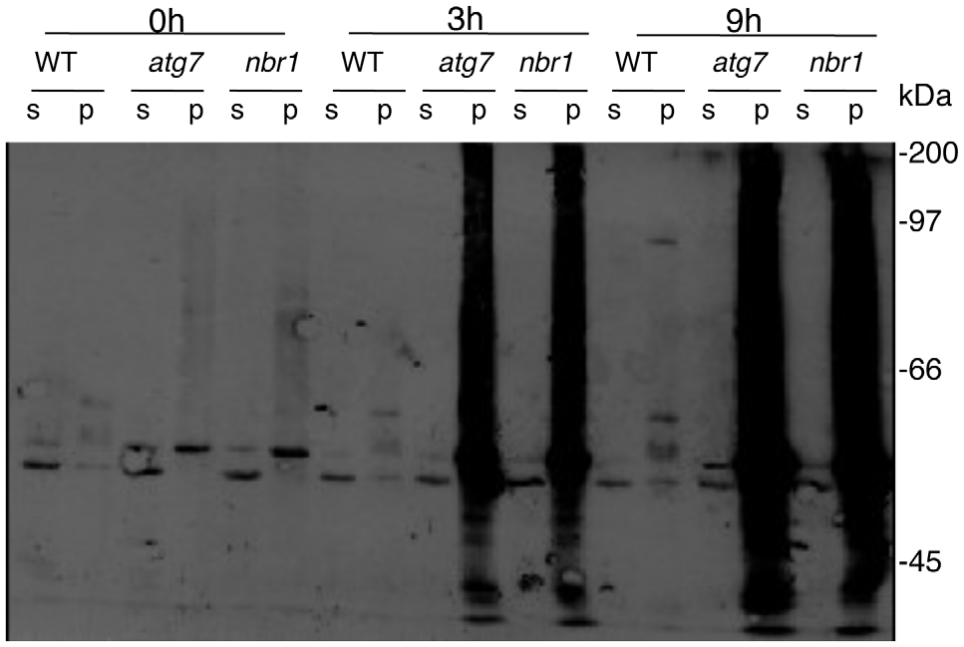
Ubiquitination of insoluble protein aggregates in the *atg7* and *nbr1* mutants under heat stress. Leaf tissues from wild type (WT), *atg7* and nbr1 mutants were collected at indicated hours (h) under 45°C and prepared for soluble and insoluble proteins as described in Materials and Methods. Proteins from the first supernatants (S) and last pellets (P) were subjected to SDS-PAGES and probed with anti-ubiquitin monoclonal antibody. The experiment was repeated three times with similar results.

*Arabidopsis* NBR1 contains two UBA domains with the C-terminal one capable of binding ubiquitin [28] and may play a critical role in stress tolerance by targeting ubiquitinated protein aggregates that are formed under stress conditions. To test this, we compared the soluble and insoluble proteins for the levels of ubiquitination prior to and after heat stress. We isolated both soluble and insoluble proteins from the leaves of the wild type, *atg7* and *nbr1* mutants after 0, 3 and 9 hours of heat treatment. The proteins were fractionated on a SDS gel and analyzed for ubiquitinated proteins using an anti-ubiquitin monoclonal antibody. As shown in Figure 13, we observed similar levels of ubiquitinated proteins in the soluble fractions in these plants with or without heat stress. In the insoluble fractions, we observed only slighly higher levels of ubiquitinated proteins in the *atg7* and *nbr1* mutants than in wild type when grown at 22°C (Figure 13). However, after 3 hours at 45°C, we observed a drastic increase in the levels of ubiquitinated proteins in the *atg7* and *nbr1* mutants but not in the wild-type plants (Figure 13). The levels of ubiquitinated proteins were further increased in the *atg7* and *nbr1* mutants after 9 hours at 45°C (Figure 13). Thus, proteins in the insoluble fraction from heat-stressed *atg7* and *nbr1* mutants are highly ubiquitinated.

## Discussion

Autophagy plays a broad role in diverse biological processes in plants including senescence, nutrient recycling and plant immune responses. Autophagy also plays a critical role in plant responses to oxidative, drought and salt stresses. In the present study, we showed that autophagy is also involved in plant response to heat stress. First, both the formation of autophagosomes and expression of ATG genes were induced in heat-stressed *Arabidopsis* plants (Figure 2, Figure 3, Figure 4). The number of autophagosomes started to increase as early as 0.5 hour and tripled after 3 hours of heat stress at 45°C in wild-type plants (Figure 3 and Figure 4). Thus, induction of autophagosome formation in response to heat stress was quite rapid. On the other hand, transcript levels of the *ATG* genes were elevated in heat-stressed plants with relatively slow and varying kinetics. Although increased levels of transcripts for some of the *ATG* genes including *ATG7* and *ATG9* were detected as early as two hours after initiation of the heat stress, other *ATG* genes exhibited increased transcript levels after 6 hours and displayed large increases after 8–10 hours of heat stress. The large increase in *ATG* gene expression may be necessary for sustained autophagosome formation under prolonged heat stress. Second, the heat tolerance of autophagy-deficient *atg5* and *atg7* mutants was compromised based on their increased morphological symptoms associated with enhanced biochemical defects and reduced recovery after heat stress (Figure 5 and Figure 6). As a common abiotic stress with increasing importance in agriculture in many parts of the world, high temperature can be manipulated relatively easily, impacts the cells of an exposed plant not only rapidly but also uniformly and, therefore, can be a useful environmental condition for studying autophagy.

In plants, studies of the roles of autophagy in diverse biological processes have been mostly through functional analysis of the genes required for the core process of autophagosome assembly. On the other hand, little is known about the genes involved in other important processes of autophagy such as autophagy cargo recognition, sequestration and transport. As a result, our knowledge about the mechanistic basis for the broad roles of plant autophagy in diverse biological processes is very limited. In the present study, we isolated and characterized *Arabidopsis* NBR1, a homolog of mammalian autophagic adaptors P62 and NBR1. Identification and characterization of NBR1 and a similar protein from tobacco, Joka2, have also been recently reported [28], [31]. These studies suggest that plant NBR1 proteins are autophagy cargo adaptors based on the structures, interaction with ATG8, subcellular localization and autophagic degradation. To determine the biological functions of plant NBR1, we isolated two independent knockout mutants for *NBR1* and conducted a comprehensive comparison of their phenotypes with those of autophagy-deficient *atg5* and *atg7* mutants in the biological processes in which autophagy is necessary. Like autophagy-deficient *atg5* and *atg7* mutants, the *nbr1* mutants were compromised in plant tolerance to heat, salt, drought and oxidative stress (Figure 5, Figure 6, Figure 7). Mutant NBR1 with a mutated LIR motif unable to interact with ATG8 is not functional in conferring plant stress tolerance (Figure 10), indicating that the role of NBR1 in plant stress tolerance is mediated by autophagy. Unlike *atg5* and *atg7* mutants, *nbr1* mutants were normal in age- and dark-induced senescence and in response to necrotrophic pathogen infection (Figure 8 and Figure 9). Thus NBR1-mediated autophagy is necessary only in plant responses to specific abiotic stresses including heat, ROS, salt and drought but is dispensable in plant senescence, nutrient recycling and defense responses. The lack of phenotypes of *nbr1* mutants in some of the biological processes involving autophagy suggests that the broad roles of autophagy in diverse biological processes are mediated by multiple cargo recognition and delivery systems in plants.

As an autophagy adaptors, the selective roles of NBR1 in plant response to specific abiotic stresses is most likely mediated by its ability in recognizing specific cargo proteins generated under abiotic stresses and facilitating their delivery to autophagosomes for degradation. *Arabidopsis* NBR1 contains a C-terminal UBA domain capable of binding ubiquitin [28] (Figure 10A), suggesting that NBR1 recognizes ubiquitinated protein substrates during plant stress responses. To identify NBR1-captured cargo proteins, we used the TAP procedure but failed to isolate sufficient amounts of NBR1-interacting proteins from the soluble fraction of heat-stressed *atg7* mutant, in which cargo proteins are not subjected to autophagic degradation and, therefore, should accumulate to high levels. Further analyses revealed that under heat stress, NBR1 was increasingly enriched in the insoluble fraction in the *atg7* mutant (Figure 11) and the compromised heat tolerance of the *atg5*, *atg7* and *nbr1* mutants were also associated with increased accumulation of insoluble detergent-resistant protein aggregates under heat stress (Figure 12). Importantly, insoluble protein aggregates accumulated in heat-stressed *atg7* and *nbr1* mutants were highly ubiquitinated (Figure 13). Abiotic stresses such as high temperature causes damages to a variety of cellular structures and macromolecules including protein denaturation and aggregation [41]. Apparently, under heat stress, denatured or otherwise damaged cellular proteins are ubiquitinated by plant cellular protein quality control machinery and targeted by NBR1 for autophagic degradation. In autophagy-deficient mutants, these denatured or otherwise damaged proteins are still ubiquitinated but not degraded and therefore accumulate at high levels as insoluble detergent-resistant protein aggregates.

Protein ubiquitination, catalyzed by a cascade of reactions involving a ubiquitin-activating enzyme (E1), a ubiquitin-conjugating enzyme (E2) and a ubiquitin ligase (E3), plays diverse roles in regulating cellular activities including selection of proteins to be degraded [42]. The majority of the cytosolic proteins destined for degradation in the eukaryotic cells are first polyubiquitinated and then targeted for degradation by the 26S proteasomes, a large protease complex consisting of a barrel-shaped 20S proteolytic core in association with two 19S regulatory caps [42]. For degradation by the proteasomes, proteins must be unfolded to enter the 13-Å wide central cavity since the steric conditions of a folded globular protein would not fit through the narrow entrance channel [43]. Under certain stress or pathological conditions, protein substrates to be degraded may form non-dissociable aggregates and, therefore, cannot be processed by proteasomes. In fact, accumulation of protein aggregates such as the polyglutamine-expanded huntingtin, which is associated with the neurodegenerative Huntington disease, can inhibit proteasome activities by clogging the proteasomes [44]. The increased accumulation of ubiquitinated proteins in heat-stressed autophagy mutants indicates that autophagy is also a major route for degradation of ubiquitinated proteins under stress conditions in plants. Conceivably, under heat and other related stress conditions, some of the cellular proteins are irreversibly denatured or damaged and, as a result, form non-dissociable protein aggregates that can be efficiently degraded only by NBR1-mediated selective autophagy but not by size-limited proteasomes.

Direct protein quantification revealed approximately a 3-fold increase in insoluble detergent resistant proteins in the *atg7* and *nbr1* mutants over the wild-type plants after 9 hours of heat stress (Figure 12A). However, western blotting showed much higher levels of ubiquitinated proteins in the insoluble fraction of the *atg7* and *nbr1* mutants than in the wild-type plants (Figure 13). This discrepancy indicated that the insoluble proteins from the wild-type plants were much less ubiquitinated than those from the *atg7* and *nbr1* mutants. It is possible that in heat-stressed wild-type plants, denatured or otherwise damaged proteins would form insoluble aggregates and then gradually become ubiquitinated. Those ubiquitinated protein aggregates would be recognized by NBR1 and preferentially degraded, while those un-ubiquitinated or under-ubiquitinated proteins aggregates would accumulate in the insoluble fraction. In addition, some of the un-ubiquitinated proteins in the insoluble fraction might come from contamination from the soluble fraction or from proteins that were denatured or damaged during the isolation process.

Comparison of protein profiles by SDS/PAGE reveled that the most abundant soluble proteins were also present abundantly in the insoluble fraction in heat-stressed *atg* mutant (Figure 12B). Since these abundant insoluble proteins were present at much lower levels in heat-stressed wild-type plants, it is unlikely that they were resulted from contamination of soluble proteins or from protein denaturation or aggregation during the isolation process. More likely, they were derived from denatured or otherwise damaged soluble proteins during heat stress that were rapidly degraded in wild type but accumulated as insoluble protein aggregates in the autophagy-deficient mutants. Thus, it seems that under heat stress many if not all abundant soluble proteins can potentially become substrates of NBR1-mediated autophagy, presumably because a majority of plant cellular proteins are not very heat-resistant and are prone to denaturation and aggregation after a prolonged period at 45°C. Under milder heat or other stress conditions, however, plant cellular proteins may display differential proneness in aggregation and therefore may be differentially targeted by NBR1-mediated autophagy. Studies from yeast to mammalians have shown that misfolded or damaged proteins generated during protein synthesis or from post-synthetic modifications such as oxidation are recognized and ubiquitinated by cellular protein quality control machinery [45], [46], [47]. Our results strongly suggest that NBR1-mediated selective autophagy does not appear to target specific proteins; more likely it targets insoluble, aggregated forms of many if not all of plant cellular proteins.

As protein ubiquitination is an important and extensive mechanism in protein quality control, it is intriguing that the *nbr1* mutants shared only some but not all of the phenotypes of the autophagy-deficient *atg5* and *atg7* mutants. *Arabidopsis* has no additional genes encoding proteins structurally similar to NBR1 and, therefore, mechanisms other than functional redundancy are likely to be responsible for the lack of phenotypes of *nbr1* in some of the biological processes involving autophagy. Under carbon-deprivation conditions in dark, plant autophagy may participate in non-selective, bulk degradation of cellular contents, which may not necessarily require NBR1. During age-induced senescence, autophagy may also be engaged in the non-selective degradation of cellular structures and macromolecules of older leaves so that the nutrients can be re-distributed and utilized by young tissues and developing fruits and seeds. From the phenotype of increased senescence of autophagy mutants, however, the role of autophagy in plant age-induced senescence is not only about nutrient redistribution to young/growing tissues but also about increasing lifespan of older leaves. Indeed, the analysis of Arabidopsis autophagy mutants has revealed that salicylic acid biosynthesis and signaling is accelerated and fed into an amplification loop through reactive oxygen species to promote cell death during senescence [38]. Autophagy is also induced by this senescence-induced salicylic acid and ROS to operate a negative feedback loop modulating salicylic acid and ROS production most likely by selectively removing damaged organelles or macromolecules generated during senescence [38]. Likewise, necrotrophic pathogens kill host cells during early infection stages through a combined action of ROS, toxins, hydrolytic enzymes and other virulent factors [48], [49] and autophagy promotes plant resistance to necrotrophic pathogens by promoting host cell survival most likely through removal of irreversibly damaged, inactivated and other toxic cytoplasmic constituents in infected plant cells [24]. The normal phenotypes of *nbr1* in age-induced senescence and plant resistance to necrotrophic pathogens, however, might suggest that ubiquitination is not a major route of recognition of damaged proteins during senescence and innate immune responses. Alternatively irreversibly damaged and inactivated proteins are first recognized through ubiquitination during senescence and innate immune responses but are removed by other non-autophagy pathways such as 26S proteasomes or by autophagy through a different ubiquitin-recognizing adaptor. Proteomic profiling of ubiquitinated proteins under different types of stress conditions and identification of additional autophagy adaptors and their respective cargos will be very fruitful in addressing these important issues.

In summary, we isolated two *nbr1* knockout mutants and discovered that disruption of Arabidopsis *NBR1* caused increased sensitivity to a spectrum of abiotic stresses but had no significant effect on plant senescence, responses to carbon starvation or resistance to a necrotrophic pathogen. A selective role of NBR1 in plant responses to specific abiotic stresses suggest that plant autophagy in diverse biological processes operates through multiple cargo recognition and delivery systems. We have further discovered that under heat stress NBR1 was increasingly enriched in the insoluble fraction in association with increased accumulation of insoluble detergent-resistant protein aggregates that are highly ubiquitinated. These results strongly suggest that NBR1-mediated autophagy targets ubiquitinated protein aggregates most likely derived from denatured and otherwise damaged nonnative proteins generated under stress conditions.

## Materials and Methods

### *Arabidopsis* genotypes and growth conditions

The *Arabidopsis* mutants and wild-type plants used in the study are all in the Co-0 background. The *atg5* and *atg7* mutants have been previously described [23], [24]. Homozygous *nbr1-1* (Salk_135513) and *nbr1-2* (GABI_246H08) mutants were identified by PCR using primers flanking the T-DNA/transposon insertions (5′-AGCATCCTCGTCGTGTTTGT-3′ and 5′-CAACCTAACTCAAGCCATCG-3′) (Figure S2). Quantitative RT-PCR of the *NBR1* transcripts were reduced more than 20 fold in the *nbr1-1* and *nbr1-2* mutants, indicating that they are both knockout mutants (Figure S2). *Arabidopsis* plants were grown in growth chambers at 22°C, 120 µE m^−2^ light on a photoperiod of 12 h light and 12 h dark.

### BiFC Assays of ATG8a–NBR1 interaction

The ATG8a coding sequence was fused with N-YFP to generate N-terminal in-frame fusions with N-YFP, and DNA sequences for NBR1 and NBR1 W661A/I664A were fused with C-YFP to generate C-terminal in-frame fusions with C-YFP as previously described [50]. The resulting clones were verified by sequencing. The plasmids were introduced into *Agrobacterium tumefaciens* (strain GV3101), and infiltration of *N. benthamiana* was performed as described previously [50]. Infected tissues were analyzed at approximately 24 hours after infiltration. Fluorescence staining were visualized using a Zeiss LSM710 confocal microscope and images were superimposed using ZEISS LSM710 software.

### RNA isolation and quantitative RT–PCR

Total RNA was isolated from 4-week-old plants using Trizol reagent (Sangon, China), according to the manufacturer's recommendations. Genomic DNA was removed with the RNeasy Mini Kit (Qiagen, Germany). Total RNA (1 µg) was reverse-transcribed using ReverTra Ace qPCR RT Kit (Toyobo, Japan), following the manufacturer's instructions. Gene-specific RT-PCR primers were designed based on their cDNA sequences (Table S1).

Quantitative real-time PCR was performed using the iCycler iQTM real-time PCR detection system (Bio-Rad, Hercules, CA, USA). Each reaction (25 µL) consisted of 12.5 µL SYBR Green PCR Master Mix (Takara, Japan), 1 µL of diluted cDNA and 0.1 µmol of forward and reserve primers. PCR cycling conditions were as follows: 95°C for 3 min, and 40 cycles of 95°C for 10 seconds (s) and 58°C for 45 s. The relative gene expression was calculated as previously described [51]. The *Arabidopsis ACTIN2* gene was used as internal control as previously described [52].

### Visualization of induction of autophagy using GFP-ATG8a and NBR1-GFP

Transgenic wild-type Col-0 plants expressing a GFP–ATG8a fusion construct were previously described [24]. To generate transgenic *atg7-2* mutant plants expressing GFP–ATG8a, the fusion construct was transformed into *atg7-2* using the floral-dip method [53] and transgenic plants were identified on the basis of kanamycin resistance and confirmed by RNA blotting using the GFP DNA fragment as a probe.

For generating transgenic *NBR1-GFP* plants, *Arabidopsis NBR1* full-length cDNA was PCR-amplified using gene-specific primers (5′-CGATGGAGTCTACTGCTAACGCA-3′ and 5′- GAAGAAGAGAGGTGCTGCCATGGCAGCCTCCTTCTCCCCTGTGAG-3′) and fused to a GFP gene. The *NBR1–GFP* fusion gene was sub-cloned under the control of the *CaMV 35S* promoter in the PFGC5941 binary vector. Transgenic plants were identified on the basis of Basta resistance, and confirmed by RNA blotting using a GFP DNA fragment as probe.

For visualization of induction of autophagy, 4-weeks old transgenic plants expressing the *GFP-ATG8a* and *GFP-NBR1* fusion gene were treated with or without heat shock for 3 h and recovered for 0.5 hour. The leaves of transgenic plants were observed using LSM710 confocal microscope with excitation at 488 nm, and imageswere superimposed using ZEISS LSM710 software.

### Analysis of abiotic stress tolerance

For testing heat tolerance, five weeks-old *Arabidopsis* Col-0 wild type (WT) and mutant plants were placed in 22°C and 45°C growth for 10 hours and then immediately analyzed for electrolyte leakage (EL) or *Fv/Fm*, or moved to room temperature for 3–5 day recovery for observation of heat stress symptoms. For testing tolerance to oxidative stress, five weeks-old *Arabidopsis* plants were sprayed with 20 µM methyl viologen (MV) and kept under light for two days before the picture of representative plants was taken. For testing drought tolerance, five weeks-old *Arabidopsis* plants were placed into a growth chamber with approximately 50% humidity. The plants were unwatered and observed for drought stress symptom development. For testing salt tolerance, seven days-old seedlings grown on solid MS medium were transferred to the same medium with or without added NaCl (0.16 M) and the survived seedlings were scored 5 days after the transfer.

### Determination of electrolyte leakage in leaves

For determination of electrolyte leakage caused by high temperature, the leaves of 4 to 5-week-old plants were measured after different treatments as previously described [54].

### Analysis of chlorophyll fluorescence

Chlorophyll (chl) fluorescence was measured using an Imaging-PAM Chlorophyll Fluorometer equipped with a computer-operated PAM-control unit (IMAG-MAXI; Heinz Walz, Effeltrich, Germany). The seedlings were kept in the dark for approximately 30 min before the measurements were taken. The intensities of the actinic light and saturating light settings were 280 µmol mol^−2^ s^−1^ and 2500 µmol mol^−2^ s^−1^ PAR, respectively. The maximum quantum yield of PSII (*Fv/Fm*) were measured and calculated as previously described [52].

### Generation of transgenic *NBR1* lines

For generating transgenic *NBR1* over-expression lines, the full-length coding sequences for *NBR1* genes was first PCR-amplified using gene-specific primers (5′- GCACAAGAAGGTCCATGGAGTCTACTGCTAACGCA-3′ and 5′-AGCTTAATTAAAGCCTCCTTCTCCCCTGTGAG-3′) and then inserted behind the *CAMV 35S* promoter in the plant transformation vector PFGC5941-myc. The NBR1W661A/I664A mutant gene was generated using the QuikChange kit from Agilent with the following primers: 5′-GAGTTAGCGAGGCGGATCCAGCCCTAGAGGAGCT-3′ and 5′-AGCTCCTCTAGGGCTGGATCCGCCTCGCTAACTC-3′. The resulted plasmids were transformed into *nbr1-1* mutant plants and transformants were identified for resistance to Basta. Transgenic plants overexpressing the *NBR1* transgene were identified by western blot using an anti-myc monoclonal antibody (Figure S2). Homozygous T2 transformants were used in the study.

### *Botrytis* infection

Culture and inoculation of *B. cinerea* were performed as previously described [24], [55]. Biomas of the fungal pathogen was quantified by qRT-PCR of total RNA isolated from inoculated plants for the *B. cinerea ActA* gene transcript levels as described previously [55].

### Generation of transgenic NBR1-TAP plants

Full-length *NBR1* cDNA was PCR-amplified using gene-specific primers (5′- GCACAAGAAGGTCCATGGAGTCTACTGCTAACGCA -3′ and 5′- GAAGAAGAGAGGTGCTGCCATGGCAGCCTCCTTCTCCCCTGTGAG - 3′) and then inserted behind the*CAMV 35S* promoter in the plant transformation/TAP vector [56]. The resulted plasmid were transformed into Col-0 and *atg7-2* mutants. Transformants were identified for resistance to Gentamycin. Transgenic plants expressing similar levels of the *NBR1* transgene were identified by northern blotting.

### Separation of soluble and insoluble proteins and Western blotting

*Arabidopsis* leaves were collected before and after heat treatment, ground in liquid nitrogen and homogenized in an detergemt-containing extraction buffer (100 mMTris/HCl, pH 8.0, 10 mM NaCl, 1 mM EDTA, 1% Triton X-100, 0.2% ß-mercaptoethanol). The homogenates were filtered through a 300 µm and 100 µm nylon mesh and clarified by centrifugation at 2,200xg for 5 minutes. Supernatants were kept for further analysis. The pellets were resuspended in the same buffer and subjected to the low speed centrifugation. The process was repeated twice and after the last centrifugation the pellets were resuspended in the extraction buffer. The concentrations of proteins in the homogenates (total proteins), the first supernatants (soluble proteins) and last pellets (insoluble proteins) were determined using Bio-Rad protein assay kit. The first supernatant fractions and last pellets were separated by SDS–PAGE. For western blotting, fractionated proteins on SDS/PAGE gel were transferred to nitrocellulose membrane. NBR1-TAP was detected by a peroxidase-conjugated anti-peroxidase antibody. Ubiquitinated proteins were detected by protein blotting using an anti-ubiquitin monoclonal antibody (Sigma, USA). The antigen-antibody complexes were detected by enhanced chemiluminescence using luminal as substrate as previously described [39].

### Accession numbers

Sequence data for the genes described in this study can be found in the GenBank/EMBL data libraries under the accession numbers shown in parentheses: *ACTIN2* (AT3G18780), *ATG5* (At5g17290), *ATG6* (At3g61710), *ATG7* (At5g45900), *ATG8a* (AT4G21980), *ATG9* (At2g31260), *ATG10* (At3g07525), *ATG18a* (At3g62770), *NBR1* (AT4G24690).

## Supporting Information

Figure S1 Induction of *ATG8* genes by heat stress. Five-week-old *Arabidopsis* wild-type Col-0 plants were placed in a 45°C growth chamber and total RNA was isolated from leaf samples collected at 0 and 10 hours of heat stress. Transcript levels were determined using real-time qRT-PCR and fold induction of an *ATG8* gene by heat stress was calculated from the ratio of the transcript levels from heat-stressed plants over those from control plants. Error bars indicate SE (n = 3).
(PPTX)

Figure S2 Structure and mutants for the *NBR1* gene. (A) Exon and intron structure of *NBR1*. The exons are indicated with rectangles and the introns with lines. The location of T-DNA insertions for the *nbr1-1* and *nbr1-2* mutants as well as the two primers for PCR genotyping of the mutants (P1 and P2) are indicated. (B) PCR identification of homozygous *nbr1* mutants. An 816 bp DNA fragment was amplified from Col-0 wild type (WT) but not from the homozygous *nbr1* mutant plants using primers flanking the T-DNA insertion sites. (C) Transcript levels of *NBR1* in WT and *nbr1* mutants as determined using real-time qRT-PCR.
(PPT)

Figure S3 Western blotting analysis of transgenic plants expressing myc-tagged *NBR1* or *mNBR1* transgene. Total proteins were extracted from the leaves and equal amounts of proteins were subjected to SDS-PAGES, probed with an anti-myc monoclonal antibody or stained with Coomassie brilliant blue.
(PPT)

Figure S4 RNA blotting analysis of transgenic plants expressing *NBR1-TAP*. Total RNA was isolated from homozygous F3 progeny of transgenic plants expressing the NBR1-TAP transgene under the CaMV 35S promoter (35S:NBR1-TAP) in the wild type (WT) or *atg7* mutant background. RNA blot was probed with an *NBR1* gene probe. Non-transgenic wild type and *atg7* mutant were also included as control. Ethidium bromide staining of rRNA is shown for the assessment of equal loading.
(PPT)

Table S1 Primers for qRT–PCR.
(DOCX)
