# Correction: NBR1-Mediated Selective Autophagy Targets Insoluble Ubiquitinated Protein Aggregates in Plant Stress Responses

**DOI:** 10.1371/journal.pgen.1004477

**Published:** 2014-06-06

**Authors:** The PLOS Genetics Staff

Figure 3 is incorrect. NBR1-GFP images were used as GFP-ATG8a in panel A. The corrected version is provided here. This error does not affect the interpretation of the results or conclusions of the paper. The authors apologise for any confusion this may have caused. The legend is correct and has not changed.

**Figure 3:**
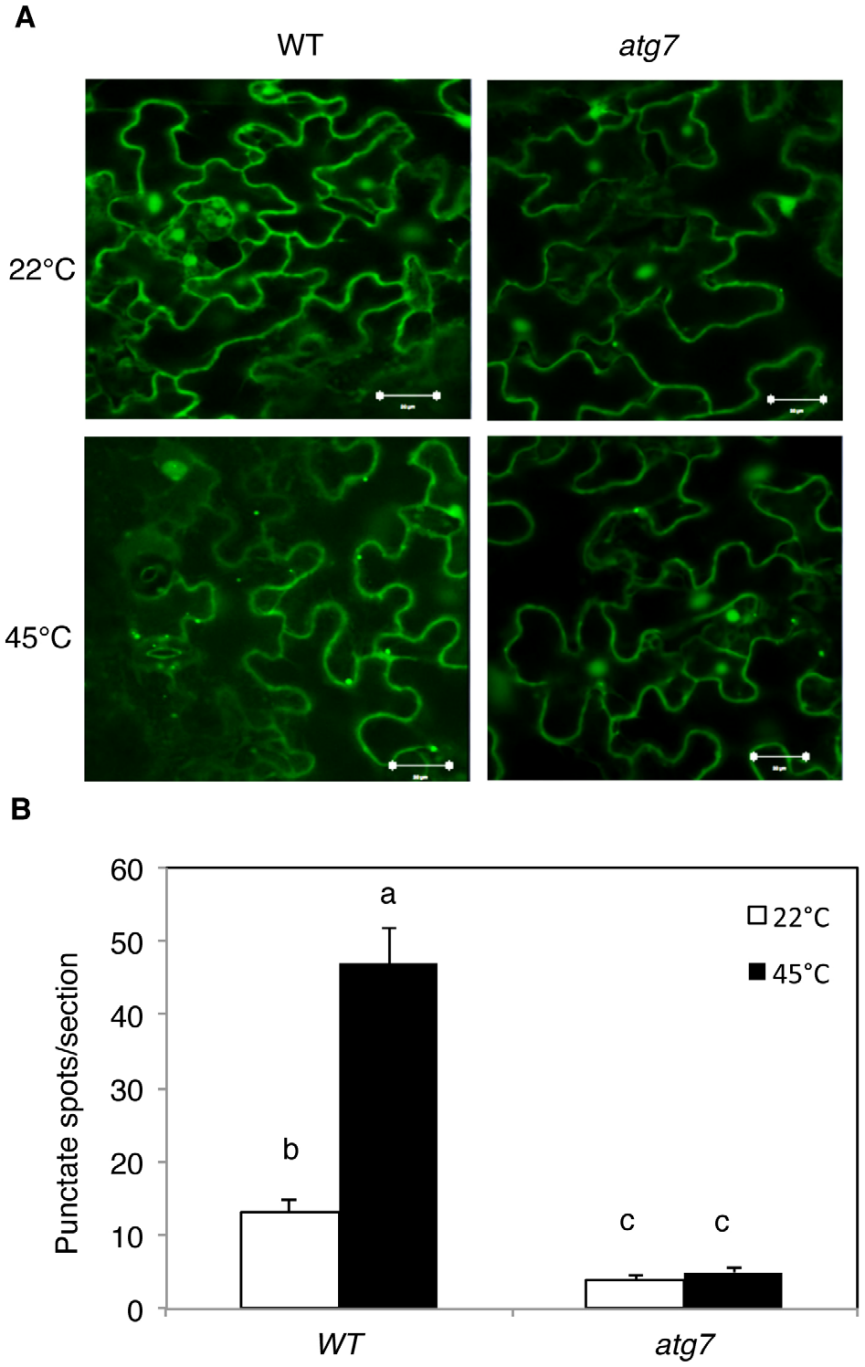
Determination of accumulation of autophagosomes using GFP-ATG8a. (A) Four-weeks old transgenic wild-type Col-0 (WT) and *atg7-2* mutant plants expressing GFP-ATG8a were treated with (45°C) or without (22°C) heat shock for 3 h and then placed at room temperature for 0.5 h. The leaves were visualized by fluorescence confocal microscopy of GFP signal. (B) Numbers of punctate GFP-ATG8a spots representing autophagosomes per 10,000 µm^2^section. Means and SE were calculated from three experiments. According to Duncan's multiple range test (P  =  0.05), means do not differ significantly if they are indicated with the same letter. Bar  =  20 µm.
