# Supplementary figures and images for: NBR1-Mediated Selective Autophagy Targets Insoluble Ubiquitinated Protein Aggregates in Plant Stress Responses
Source: PLoS Genet. 2013 Jan 17;9(1):e1003196. doi: 10.1371/journal.pgen.1003196 (PMC3547818; doi:10.1371/journal.pgen.1003196)

## Slide 1
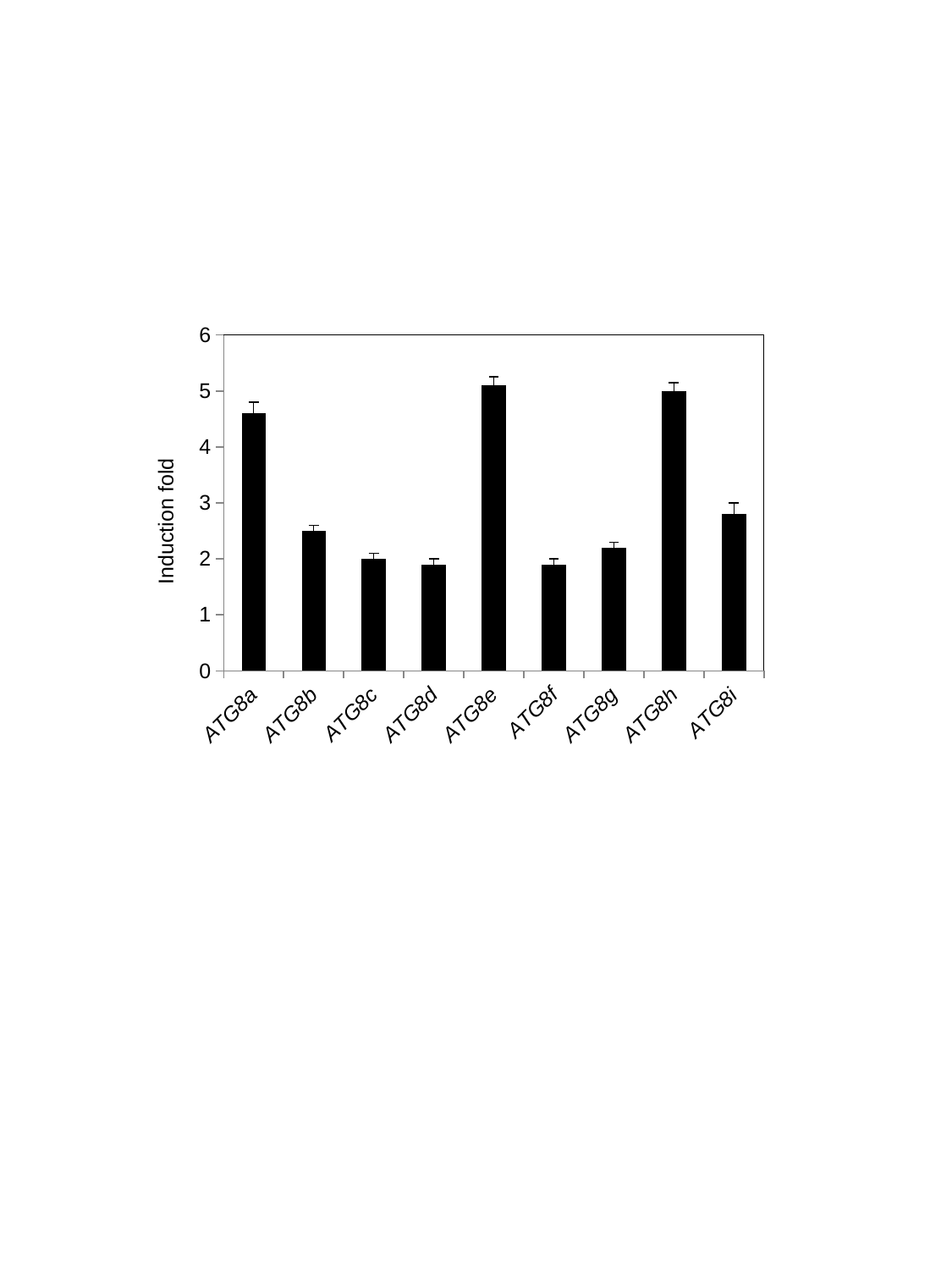

### Chart
| Category | |
|---|---|
| ATG8a | 4.6 |
| ATG8b | 2.5 |
| ATG8c | 2.0 |
| ATG8d | 1.9 |
| ATG8e | 5.1 |
| ATG8f | 1.9 |
| ATG8g | 2.2 |
| ATG8h | 5.0 |
| ATG8i | 2.8 |Induction fold

Supplement: Figure S1 — Induction of ATG8 genes by heat stress. Five-week-old Arabidopsis wild-type Col-0 plants were placed in a 45°C growth chamber and total RNA was isolated from leaf samples collected at 0 and 10 hours of heat stress. Transcript levels were determined using real-time qRT-PCR and fold induction of an ATG8 gene by heat stress was calculated from the ratio of the transcript levels from heat-stressed plants over those from control plants. Error bars indicate SE (n = 3). (PPTX) [file pgen.1003196.s001.pptx]

## Slide 1
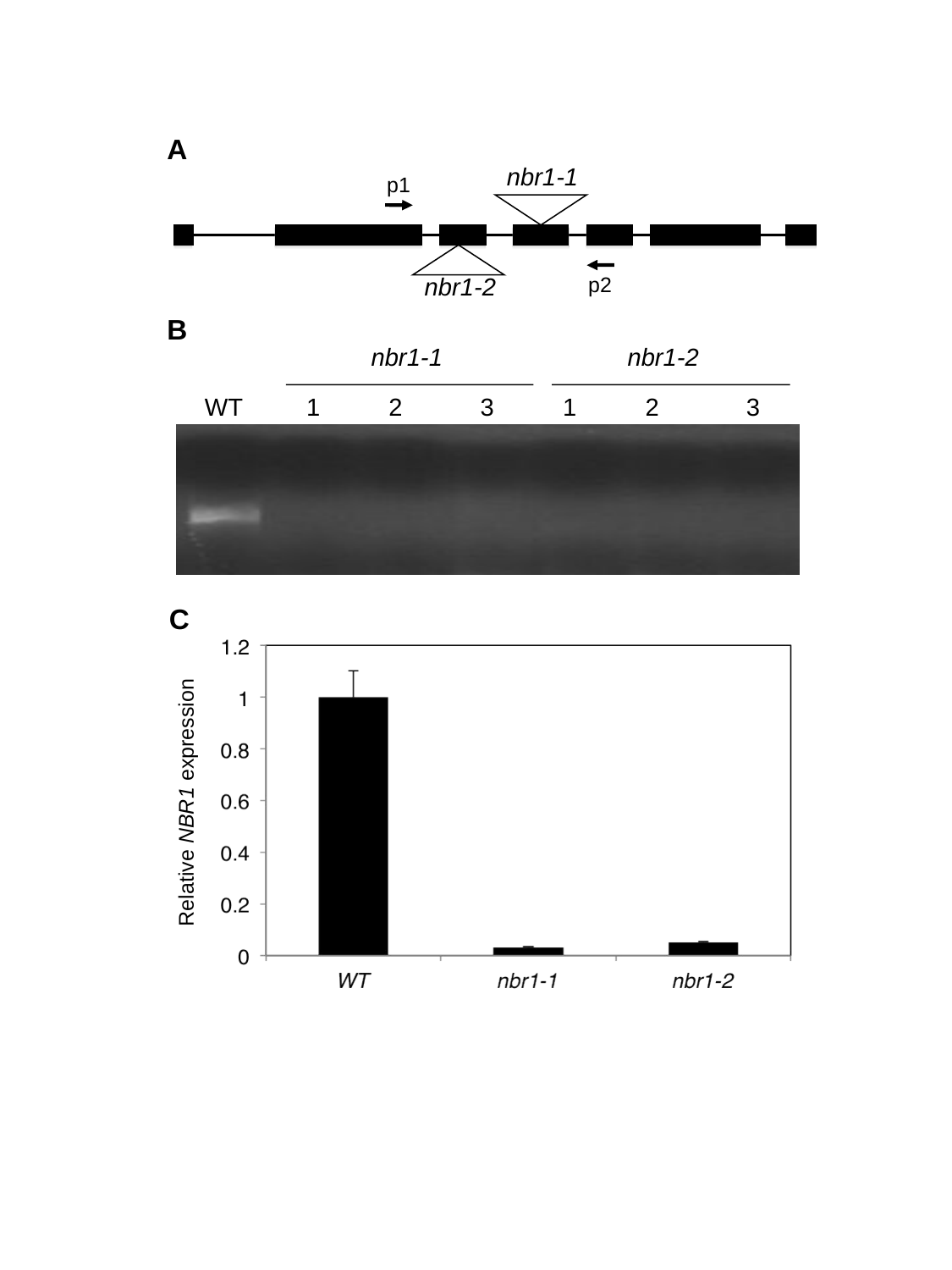

A
nbr1-1
p1
nbr1-2
p2
B
nbr1-1
nbr1-2
WT
1
2
3
1
2
3
C
Relative NBR1 expression

Supplement: Figure S2 — Structure and mutants for the NBR1 gene. (A) Exon and intron structure of NBR1. The exons are indicated with rectangles and the introns with lines. The location of T-DNA insertions for the nbr1-1 and nbr1-2 mutants as well as the two primers for PCR genotyping of the mutants (P1 and P2) are indicated. (B) PCR identification of homozygous nbr1 mutants. An 816 bp DNA fragment was amplified from Col-0 wild type (WT) but not from the homozygous nbr1 mutant plants using primers flanking the T-DNA insertion sites. (C) Transcript levels of NBR1 in WT and nbr1 mutants as determined using real-time qRT-PCR. (PPT) [file pgen.1003196.s002.ppt]

## Slide 1
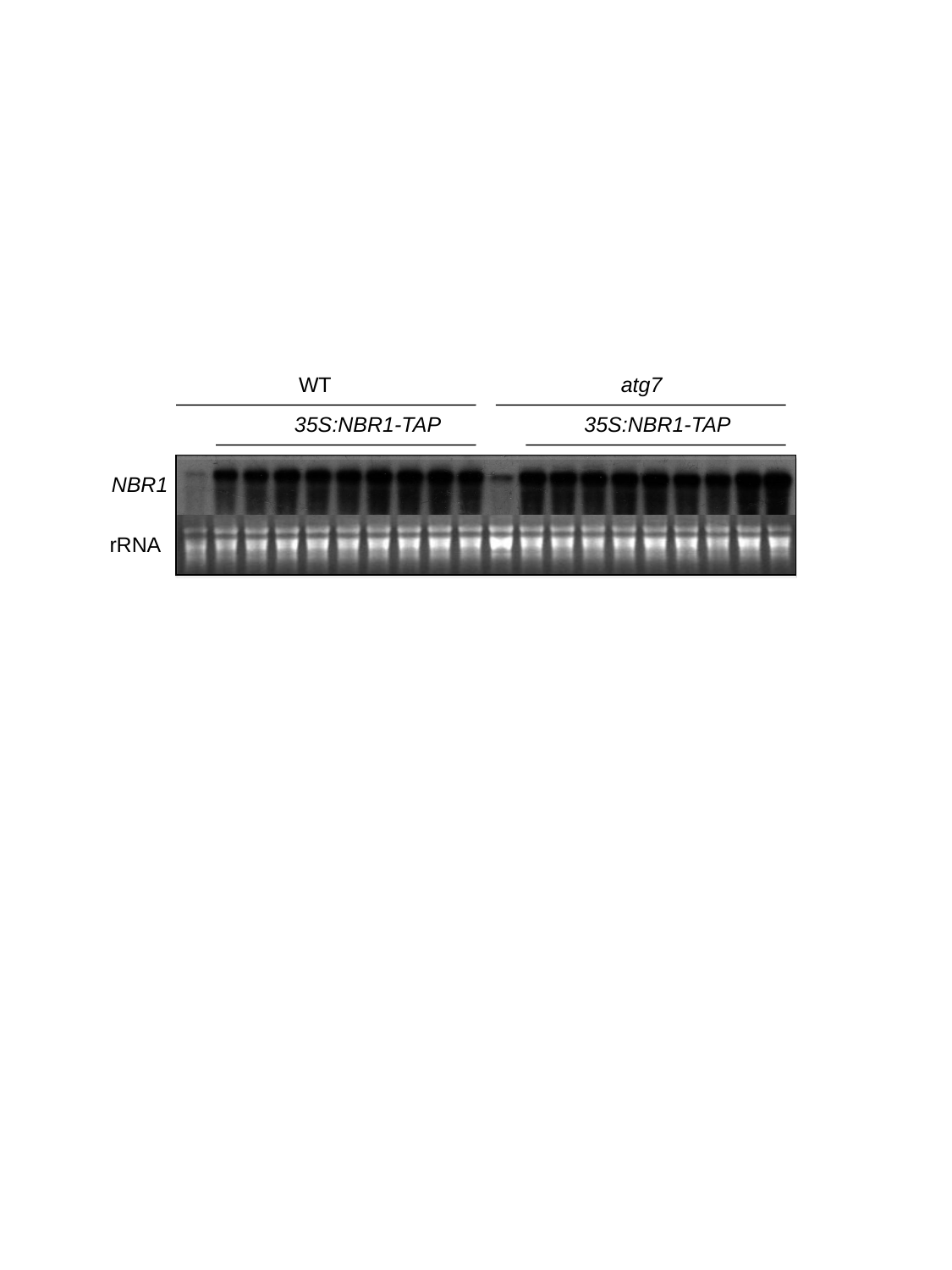

WT
atg7
35S:NBR1-TAP
35S:NBR1-TAP
NBR1
rRNA

Supplement: Figure S4 — RNA blotting analysis of transgenic plants expressing NBR1-TAP. Total RNA was isolated from homozygous F3 progeny of transgenic plants expressing the NBR1-TAP transgene under the CaMV 35S promoter (35S:NBR1-TAP) in the wild type (WT) or atg7 mutant background. RNA blot was probed with an NBR1 gene probe. Non-transgenic wild type and atg7 mutant were also included as control. Ethidium bromide staining of rRNA is shown for the assessment of equal loading. (PPT) [file pgen.1003196.s004.ppt]
