# Supplementary material for: NBR1-Mediated Selective Autophagy Targets Insoluble Ubiquitinated Protein Aggregates in Plant Stress Responses
Source: PLoS Genet. 2013 Jan 17;9(1):e1003196. doi: 10.1371/journal.pgen.1003196 (PMC3547818; doi:10.1371/journal.pgen.1003196)
Supplement: Figure S3 — Western blotting analysis of transgenic plants expressing myc-tagged NBR1 or mNBR1 transgene. Total proteins were extracted from the leaves and equal amounts of proteins were subjected to SDS-PAGES, probed with an anti-myc monoclonal antibody or stained with Coomassie brilliant blue. (PPT) [file pgen.1003196.s003.ppt]

## Slide 1
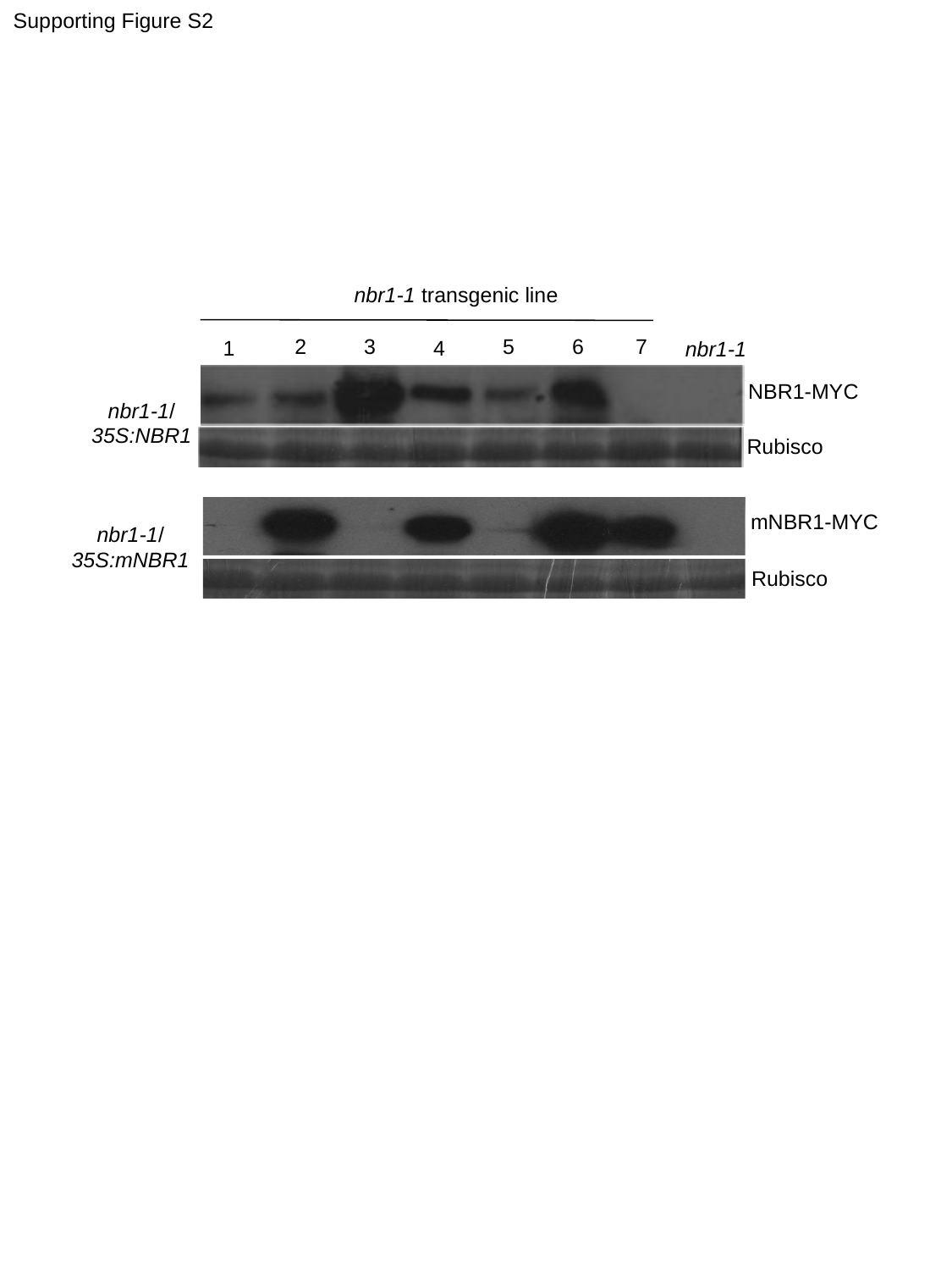

Supporting Figure S2
nbr1-1 transgenic line
2
3
5
6
7
1
4
nbr1-1
NBR1-MYC
nbr1-1/
35S:NBR1
Rubisco
mNBR1-MYC
Rubisco
nbr1-1/
35S:mNBR1
