# Supplementary material for: NBR1-Mediated Selective Autophagy Targets Insoluble Ubiquitinated Protein Aggregates in Plant Stress Responses
Source: PLoS Genet. 2013 Jan 17;9(1):e1003196. doi: 10.1371/journal.pgen.1003196 (PMC3547818; doi:10.1371/journal.pgen.1003196)
Supplement: Table S1 — Primers for qRT–PCR. (DOCX) [file pgen.1003196.s005.docx]

Supporting Table S1: Primers for qRT-PCR

| GENE | GI | PRIMERS | |
| --- | --- | --- | --- |
|  |  | FORWARD | REVERSE |
| Actin2 | AT3G18780 | GCTGACCGTATGAGCAAAGA | ATCTGCTGGAATGTGCTGAG |
| ATG5 | AT5G17290 | ATGGGAACAGTCGAAGATGA | ACGAGATGTCATCCCAGGTA |
| ATG6 | AT3G61710 | AGCCATTGTGCTTAGAATGC | CCTTCTAACCTCTGAACGCA |
| ATG7 | AT5G45900 | TCTATGACCCGTGTCACCTT | GAGGCTTGACCAACAAGAGA |
| ATG8a | AT4G21980 | CAAGCTTGGAGCTGAGAAAG | GCAACGGTAAGAGATCCAAA |
| ATG8b | AT4G04620 | GAATCAAGCTTGGAGCTGAA | TGTAGAGAAACCCGTCTTCG |
| ATG8c | AT1G62040 | CTTTCAAGTTGGAACACCCA | CATACACAAATTGCCCAACA |
| ATG8d | AT2G05630 | TATGTTGTACGGAAGCGGAT | AGAAACCCGTCTTCGTCTTT |
| ATG8e | AT2G45170 | GTACCTTGTGCCATCAGACC | AGGAAGCCATCTTCGTCTTT |
| ATG8f | AT4G16520 | AGTACCTAGTCCCGGCTGAT | AGGAAGAACATTGTCCACGA |
| ATG8g | AT3G60640 | TTCGAGAAGAGGAAAGCTGA | TTAGATCAGCTGGGACAAGG |
| ATG8h | AT3G06420 | ATCTGCCAGACATGGAGAAG | CAAAGAGAGCTTTGGATGGA |
| ATG8i | AT3G15580 | GTCAGTTGGCCAATTCATCT | GTCCATCAGAGCAGCAGTTT |
| ATG9 | AT2G31260 | TGGGAAGAGAATGCAAGAAG | ACCGTAATGTGGTGCTTGAT |
| ATG10 | AT3G07525 | CATGGTTCAAGCTACATCCC | GGGATCCTAAGACCAACCAC |
| ATG18a | AT3G62770 | TGTTTCTCAGGGTGTTGGTT | TGAGAGCGAAGCAAGCTATT |
| NBR1 | AT4G24690 | GAGGAAATGGGTTTCAAGGA | ATTGGATCCCACTCGCTAAC |
